# Supplementary material for: Limited Evidence for the Benefits of Exercise in Older Adults with Hematological Malignancies: A Systematic Review and Meta-Analysis
Source: Cancers (Basel). 2024 Aug 25;16(17):2962. doi: 10.3390/cancers16172962 (PMC11393877; doi:10.3390/cancers16172962)

Figure S1. Search strategy and PRISMA flow diagram for study selection (March 2019), study population above 65 years.

Four electronic databases were searched in March 2019: MEDLINE, EMBASE, CINAHL and CENTRAL. The search matrix had three search elements: 1) exercise-based interventions; 2) hematological malignancies in medical antineoplastic treatment; and 3) randomized controlled trials, and the first two for CENTRAL. A combination of MeSH/Thesaurus/indexed terms and relevant synonyms for the first two search elements were used. Randomized controlled trials were identified by the Cochrane Collaboration highly sensitive search strategy for identifying randomized controlled trials. Study population $\geq$ 65 years included. No restrictions were applied for language or time of publication. A total of n= 4096 hits. Duplicates (across datasets) were removed automatically in EndNote prior to transferring to Covidence, resulting in 3458 studies being imported to Covidence (see flowchart below). MJ og CG screened title/abstracts, 47 full text screening. Resulted in 0 studies.

| **Database** | **Number of documents** |
|---|---|
| MEDLINE | 2008 |
| EMBASE | 1553 |
| CINAHL | 130 |
| CENTRAL | 405 |
| **Total** | **4096** |
| *Imported to Covidence after initial removal of duplicates | 3458 |

**PubMed MEDLINE 06-03-2019**

#1 Myeloma*[tiab] OR Haematologic cancer*[tiab] OR Hematologic cancer*[tiab] OR Haematological cancer*[tiab] OR Hematological cancer*[tiab] OR Haematologic malignanc*[tiab] OR Hematologic malignanc*[tiab] OR Haematological malignanc*[tiab] OR Hematological malignanc*[tiab] OR Hematologic Neoplasms[mh] OR Haematologic neoplasm*[tiab] OR Hematologic neoplasm*[tiab] OR Haematological neoplasm*[tiab] OR Hematological neoplasm*[tiab] OR Leukaemia*[tiab] OR Leukemia[mh] OR Leukemia*[tiab] OR Lymphoma[mh] OR Lymphoma*[tiab]

#2 "Cardiovascular training"[tiab] OR Sport*[tiab] OR Sports[mh] OR Crossfit[tiab] OR Danc*[tiab] OR Dancing[mh] OR "Endurance training"[tiab] OR Exercise Movement Techniques[mh] OR Exercise[tiab] OR Exercise[mh] OR Exercise Therapy[mh] OR Fitness[tiab] OR Jogg*[tiab] OR Kinesiotherap*[tiab] OR Martial art*[tiab] OR Martial Arts[mh] OR "Muscle strengthening"[tiab] OR "Muscle training"[tiab] OR "Strength training"[tiab] OR "Resistance training"[tiab] OR "Weight lifting"[tiab] OR Physical activit*[tiab] OR Pilates[tiab] OR Yoga[tiab] OR Running[tiab] OR Step activit*[tiab] OR "Tai chi"[tiab] OR "Tai ji"[tiab] OR Walk*[tiab] OR "High-intensity interval training"[tiab] OR "Physical Therapy Modalities"[mh]

#3 (randomized controlled trial[pt] OR controlled clinical trial[pt] OR randomized[tiab] OR placebo[tiab] OR drug therapy[sh] OR randomly[tiab] OR trial[tiab] OR groups[tiab]) NOT (animals [mh] NOT humans [mh])

#1 AND #2 = **2008**

#1 AND #2 AND #3 = 588

#3 = Sensitivity-maximizing version (2008 revision); PubMed format - https://work.cochrane.org/pubmed

**EMBASE 14-03-2019**

#1 Myeloma*.ti,ab,kw. OR exp Myeloma/ OR Haematologic cancer*.ti,ab,kw. OR Hematologic cancer*.ti,ab,kw. OR Haematological cancer*.ti,ab,kw. OR Hematological cancer*.ti,ab,kw. OR Haematologic malignanc*.ti,ab,kw. OR Hematologic malignanc*.ti,ab,kw. OR exp Hematologic malignancy/ OR Haematologic neoplasm*.ti,ab,kw. OR Hematologic neoplasm*.ti,ab,kw. OR Haematological neoplasm*.ti,ab,kw. OR Hematological neoplasm*.ti,ab,kw. OR Leukaemia*.ti,ab,kw. OR Leukemia*.ti,ab,kw. OR exp Leukemia/ OR Lymphoma*.ti,ab,kw. OR exp Lymphoma/

#2 Cardiovascular training.ti,ab,kw. OR Sport*.ti,ab,kw. OR exp Sport/ OR Crossfit.ti,ab,kw. OR Danc*.ti,ab,kw. OR Endurance training.ti,ab,kw. OR Exercise movement technique*.ti,ab,kw. OR exp Exercise/ OR Exercise.ti,ab,kw. OR Fitness.ti,ab,kw. OR Jogg*.ti,ab,kw. OR exp Kinesiotherap/ OR Kinesiotherap*.ti,ab,kw. OR Martial art*.ti,ab,kw. OR exp Martial arts/ OR Muscle strengthening.ti,ab,kw. OR Muscle training.ti,ab,kw. OR Strength training.ti,ab,kw. OR Resistance training.ti,ab,kw. OR Weightlifting.ti,ab,kw. OR Physical activit*.ti,ab,kw. OR exp Physical activity/ OR Pilates.ti,ab,kw. OR Yoga.ti,ab,kw. OR Running.ti,ab,kw. OR Step activit*.ti,ab,kw. OR Tai chi.ti,ab,kw. OR Tai ji.ti,ab,kw. OR Walk*.ti,ab,kw. OR High-intensity interval training.ti,ab,kw. OR Physical Therapy Modalities.ti,ab,kw.
#3 crossover procedure/ OR double-blind procedure/ OR randomized controlled trial/ OR single-blind procedure/ OR (random* OR factorial* OR crossover* OR cross ADJ1 over* OR placebo* OR doubl* ADJ1 blind* OR singl* ADJ1 blind* OR assign* OR allocat* OR volunteer*).sh,ab,ti.
#4 random*.ab,ti. OR (clinical ADJ1 trial*).sh,ab,ti. OR exp health care quality/
#1 AND #2 = 5365
#1 AND #2 AND #3 = 359
#1 AND #2 AND #4 = 1471
(#1 AND #2 AND #4) NOT (#1 AND #2 AND #3) = 1194
(#1 AND #2 AND #3) NOT (#1 AND #2 AND #4) = 82
#1 AND #2 AND (#3 OR #4) = **1553**
#3 = Embase search strategy for finding RCTs in Embase (Lefebvre et al. 2011) - https://work.cochrane.org/embase
#4 = Alternative Embase strategies, sensitivity maximizing strategy (Wong et al. 2006) - https://work.cochrane.org/embase

**CINAHL 22-03-2019**
#1 TI Myeloma* OR AB Myeloma* OR TI Haematologic* cancer* OR AB Haematologic* cancer* OR TI Hematologic* cancer* OR AB Hematologic* cancer* OR TI Haematologic* malignanc* AB Haematologic* malignanc* OR TI Hematologic* malignanc* OR AB Hematologic* malignanc* OR MH Hematologic Neoplasms+ OR TI Haematologic* neoplasm* OR AB Haematologic* neoplasm* OR TI Hematologic neoplasm* OR AB Hematologic* neoplasm* OR TI Leukaemia* OR AB Leukaemia* OR MH Leukemia+ OR TI Leukemia* OR AB Leukemia* OR MH Lymphoma+ OR TI Lymphoma* OR AB Lymphoma*
#2 TI Cardiovascular training OR AB Cardiovascular training OR MH Sports+ OR TI Sport* OR AB Sport* OR TI Crossfit OR AB Crossfit OR TI Danc* OR AB Danc* OR MH Dancing+ OR TI Endurance training OR AB Endurance training OR MH Exercise+ OR TI Exercise OR AB Exercise OR MH Therapeutic Exercise+ OR TI Fitness AB Fitness OR TI Jogg* OR AB Jogg* OR TI Kinesiotherap* OR AB Kinesiotherap* OR MH Martial arts OR TI Martial Art* AB Martial Art* OR TI Muscle strengthening OR AB Muscle strengthening OR TI Muscle training OR AB Muscle training OR TI Strength training OR AB Strength training OR TI Resistance training OR AB Resistance training OR TI Weight lifting OR AB Weight lifting OR MH Physical activity OR TI Physical activit* OR AB Physical activit* OR TI Pilates OR AB Pilates OR MH Yoga OR TI Yoga OR AB Yoga OR TI Running OR AB Running OR TI Step activit* OR AB Step activit* OR TI Tai chi OR AB Tai chi OR TI Tai ji OR AB Tai ji OR MH Walking+ OR TI Walk* OR AB Walk* OR TI High-intensity interval training OR AB High-intensity interval training OR MH Physical Therapy OR TI Physical Therapy OR AB Physical Therapy
#3 (MH randomized controlled trials OR MH double-blind studies OR MH single-blind studies OR MH random assignment OR MH pretest-posttest design OR MH cluster sample OR TI (randomised OR randomized) OR AB random* OR TI trial OR (MH sample size AND AB (assigned OR allocated OR control)) OR MH placebos OR PT randomized controlled trial OR AB control W5 group OR MH crossover design OR MH comparative studies OR AB cluster W3 RCT) NOT ((MH animals+ OR MH animal studies OR TI animal model*) NOT MH human)
#1 AND #2 = **130**
#1 AND #2 AND #3 = 32

#3 = Glanville et al. 2019. Development of a search filter to identify reports of controlled clinical trials ... Health Info Libr J 36(1)73-90.

**Cochrane Central Register of Controlled Trials (CENTRAL) 29-03-2019**
#1 Myeloma*:ti,ab,kw OR Haematologic cancer*:ti,ab,kw OR Hematologic cancer*:ti,ab,kw OR Haematological cancer*:ti,ab,kw OR Hematological cancer*:ti,ab,kw OR Haematologic malignanc*:ti,ab,kw OR Hematologic malignanc*:ti,ab,kw OR Haematological malignanc*:ti,ab,kw OR Hematological malignanc*:ti,ab,kw OR mh "Hematologic Neoplasms" OR Haematologic neoplasm*:ti,ab,kw OR Hematologic neoplasm*:ti,ab,kw OR Haematological neoplasm*:ti,ab,kw OR Hematological neoplasm*:ti,ab,kw OR Leukaemia*:ti,ab,kw OR mh "Leukemia" OR Leukemia*:ti,ab,kw OR mh "Lymphoma" OR Lymphoma*:ti,ab,kw
#2 "Cardiovascular training":ti,ab,kw OR Sport*:ti,ab,kw OR mh "Sports" OR Crossfit:ti,ab,kw OR Danc*:ti,ab,kw OR mh "Dancing" OR "Endurance training":ti,ab,kw OR mh "Exercise Movement Techniques" OR Exercise:ti,ab,kw OR mh "Exercise" OR mh "Exercise Therapy" OR Fitness:ti,ab,kw OR Jogg*:ti,ab,kw OR Kinesiotherap*:ti,ab,kw OR Martial art*:ti,ab,kw OR mh "Martial Arts" OR "Muscle strengthening":ti,ab,kw OR "Muscle training":ti,ab,kw OR "Strength training":ti,ab,kw OR "Resistance training":ti,ab,kw OR "Weight lifting":ti,ab,kw OR Physical activit*:ti,ab,kw OR Pilates:ti,ab,kw OR Yoga:ti,ab,kw OR Running:ti,ab,kw OR Step activit*:ti,ab,kw OR "Tai chi":ti,ab,kw OR "Tai ji":ti,ab,kw OR Walk*:ti,ab,kw OR "High-intensity interval training":ti,ab,kw OR mh "Physical Therapy Modalities"
#1 AND #2 = **405**

**Covidence Flowchart:** Of the total hits (n= 4096) and after initial removal of duplicates, 3458 studies were imported into Covidence for further screening for duplicates and relevance.

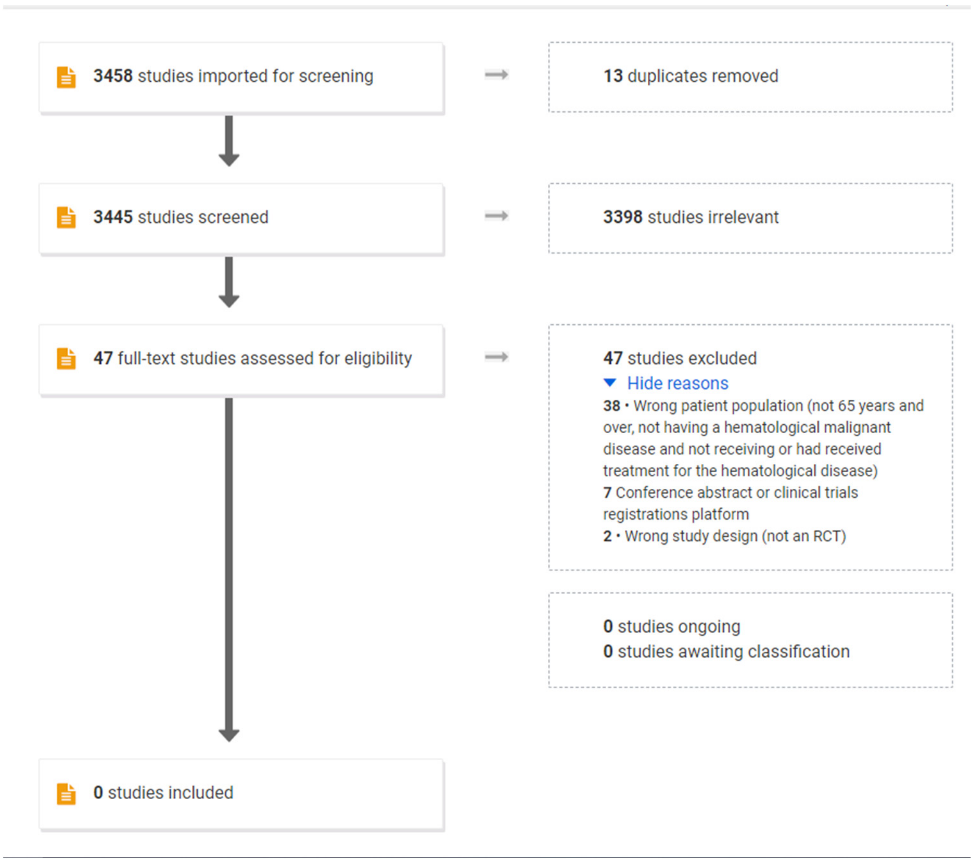

Supplement: Supplementary file 1 [file cancers-16-02962-s001.zip › Figure S1. Search strategy and PRISMA flow diagram for study selection (March 2019).pdf]
