# Supplementary material for: Limited Evidence for the Benefits of Exercise in Older Adults with Hematological Malignancies: A Systematic Review and Meta-Analysis
Source: Cancers (Basel). 2024 Aug 25;16(17):2962. doi: 10.3390/cancers16172962 (PMC11393877; doi:10.3390/cancers16172962)

Figure S20. Meta-regression analyses on exercise benefits related to age for secondary outcomes

Aerobic capacity

```
Random-effects meta-regression                    Number of obs  =       14
Method: REML                              Residual heterogeneity:
                                          tau2 =   .1683
                                        I2 (%) =   67.78
                                            H2 =   3.10
                                  R-squared (%) =     0.00
                                 Wald chi2(1)   =      1.05
                                 Prob > chi2    =    0.3059
------------------------------------------------------------------------------
  _meta_es | Coefficient  Std. err.      z    P>|z|     [95% conf. interval]
-------------+----------------------------------------------------------------
  age_mean |  -.0269303   .0263051    -1.02   0.306    -.0784873    .0246267
     _cons |   1.99846    1.337971     1.49   0.135    -.6239152    4.620835
------------------------------------------------------------------------------
Test of residual homogeneity: Q_res = chi2(12) = 42.99   Prob > Q_res = 0.0000

. estat bubble
```

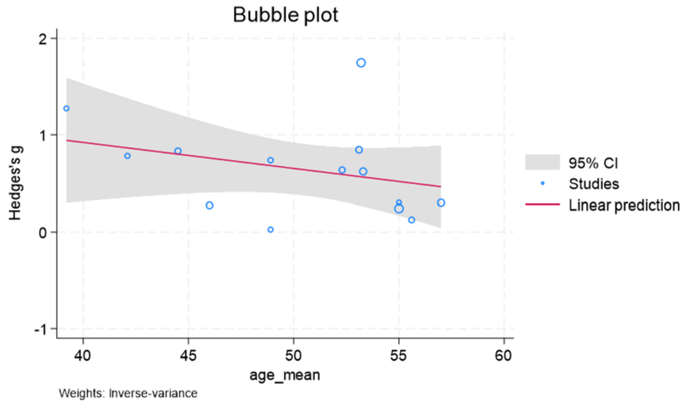

Muscle strength

```
Random-effects meta-regression                    Number of obs  =       22
Method: REML                              Residual heterogeneity:
                                          tau2 =   .5713
                                        I2 (%) =   85.65
                                            H2 =   6.97
                                  R-squared (%) =     0.00
                                 Wald chi2(1)   =      0.97
                                 Prob > chi2    =    0.3256
------------------------------------------------------------------------------
  _meta_es | Coefficient  Std. err.      z    P>|z|     [95% conf. interval]
-------------+----------------------------------------------------------------
  age_mean |  -.0208339   .0211925    -0.98   0.326    -.0623703    .0207026
     _cons |   1.514613   1.057099     1.43   0.152    -.5572619    3.586488
------------------------------------------------------------------------------
Test of residual homogeneity: Q_res = chi2(20) = 100.65  Prob > Q_res = 0.0000

. estat bubble
```

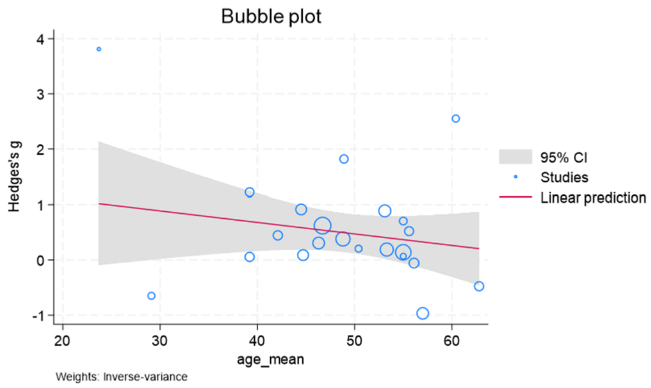

Body composition

```
Random-effects meta-regression                    Number of obs =       11
Method: REML                                  Residual heterogeneity:
                                                   tau2 =  .02647
                                                 I2 (%) =   25.51
                                                     H2 =    1.34
                                              R-squared (%) =    0.00
                                             Wald chi2(1)   =     0.17
                                             Prob > chi2    =   0.6808
------------------------------------------------------------------------------
   _meta_es | Coefficient  Std. err.      z    P>|z|     [95% conf. interval]
-------------+----------------------------------------------------------------
   age_mean |  -.0048327   .0117478    -0.41   0.681    -.0278579    .0181924
      _cons |   .4536068   .5742616     0.79   0.430    -.6719251    1.579139
------------------------------------------------------------------------------
Test of residual homogeneity: Q_res = chi2(9) = 11.69    Prob > Q_res = 0.2315

. estat bubble
```

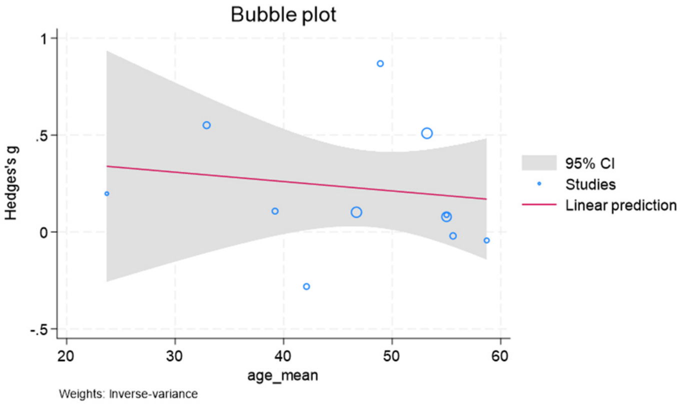

Physical activity

```
Random-effects meta-regression                    Number of obs =        5
Method: REML                                  Residual heterogeneity:
                                                   tau2 =   .1169
                                                 I2 (%) =   64.17
                                                     H2 =    2.79
                                              R-squared (%) =    0.00
                                             Wald chi2(1)   =     0.27
                                             Prob > chi2    =   0.6051
------------------------------------------------------------------------------
   _meta_es | Coefficient  Std. err.      z    P>|z|     [95% conf. interval]
-------------+----------------------------------------------------------------
   age_mean |   .0251328   .0486009     0.52   0.605    -.0701232    .1203887
      _cons |  -.9908906   2.573424    -0.39   0.700    -6.034709    4.052928
------------------------------------------------------------------------------
Test of residual homogeneity: Q_res = chi2(3) =  8.35    Prob > Q_res = 0.0392
```

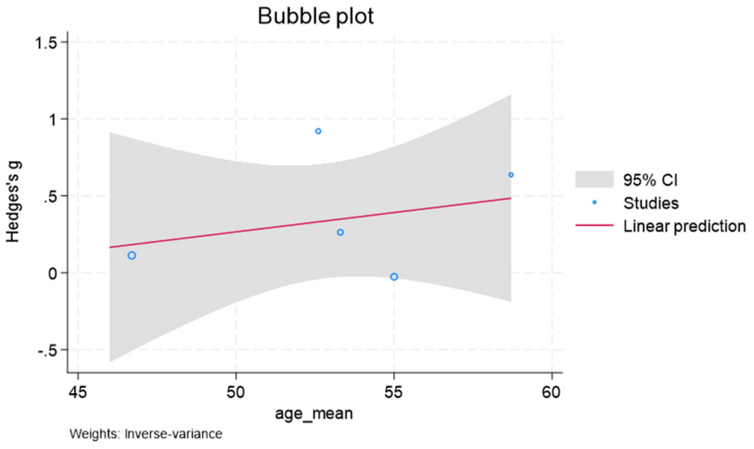

QoL emotional

```
Random-effects meta-regression                    Number of obs  =       24
Method: REML                              Residual heterogeneity:
                                                  tau2 =   .5584
                                                I2 (%) =   87.52
                                                    H2 =    8.01
                                         R-squared (%) =    0.00
                                        Wald chi2(1)   =      0.67
                                        Prob > chi2    =    0.4123
------------------------------------------------------------------------------
   _meta_es | Coefficient  Std. err.      z    P>|z|     [95% conf. interval]
-------------+----------------------------------------------------------------
   age_mean |   .0166276   .0202795     0.82   0.412    -.0231194    .0563746
      _cons |  -.5006187   1.066988    -0.47   0.639    -2.591877    1.590639
------------------------------------------------------------------------------
Test of residual homogeneity: Q_res = chi2(22) = 136.70  Prob > Q_res = 0.0000

. estat bubble
```

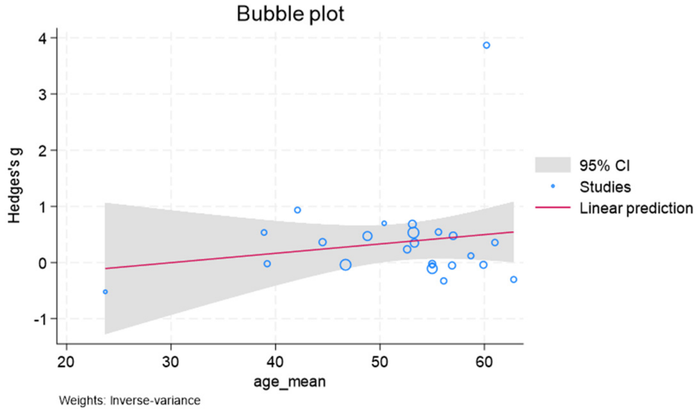

QoL functional

```
Random-effects meta-regression                    Number of obs  =        7
Method: REML                              Residual heterogeneity:
                                                  tau2 =   .1439
                                                I2 (%) =   57.32
                                                    H2 =    2.34
                                         R-squared (%) =    0.00
                                        Wald chi2(1)   =      0.00
                                        Prob > chi2    =    0.9661
------------------------------------------------------------------------------
   _meta_es | Coefficient  Std. err.      z    P>|z|     [95% conf. interval]
-------------+----------------------------------------------------------------
   age_mean |   .0007609    .017887     0.04   0.966    -.0342971    .0358188
      _cons |   .3760062   .9333934     0.40   0.687    -1.453411    2.205424
------------------------------------------------------------------------------
Test of residual homogeneity: Q_res = chi2(5) = 11.50    Prob > Q_res = 0.0422

. estat bubble
```

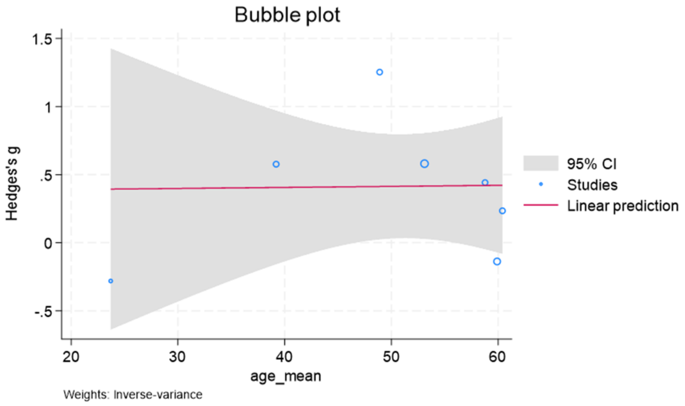

QoL physical

```
Random-effects meta-regression                    Number of obs  =       24
Method: REML                               Residual heterogeneity:
                                                  tau2 =  .5799
                                                I2 (%) =  87.89
                                                    H2 =   8.26
                                            R-squared (%) =    0.00
                                            Wald chi2(1)   =     0.71
                                            Prob > chi2    =   0.4008
------------------------------------------------------------------------------
   _meta_es | Coefficient  Std. err.     z    P>|z|     [95% conf. interval]
-------------+----------------------------------------------------------------
   age_mean |  .0173645   .0206677    0.84   0.401    -.0231434    .0578723
      _cons |  -.5106722  1.080561   -0.47   0.636    -2.628532    1.607188
------------------------------------------------------------------------------
Test of residual homogeneity: Q_res = chi2(22) = 145.09  Prob > Q_res = 0.0000

. estat bubble
```

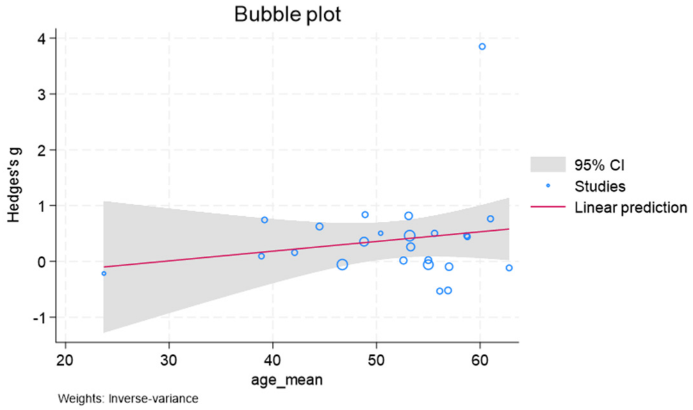

Anxiety

```
Random-effects meta-regression                    Number of obs  =       14
Method: REML                               Residual heterogeneity:
                                                  tau2 =  .5409
                                                I2 (%) =  87.04
                                                    H2 =   7.71
                                            R-squared (%) =    0.00
                                            Wald chi2(1)   =     0.60
                                            Prob > chi2    =   0.4377
------------------------------------------------------------------------------
   _meta_es | Coefficient  Std. err.     z    P>|z|     [95% conf. interval]
-------------+----------------------------------------------------------------
   age_mean |  .0263068   .033897     0.78   0.438    -.0401302    .0927437
      _cons |  -1.652302  1.769397   -0.93   0.350    -5.120257    1.815654
------------------------------------------------------------------------------
Test of residual homogeneity: Q_res = chi2(12) = 74.20   Prob > Q_res = 0.0000

. estat bubble
```

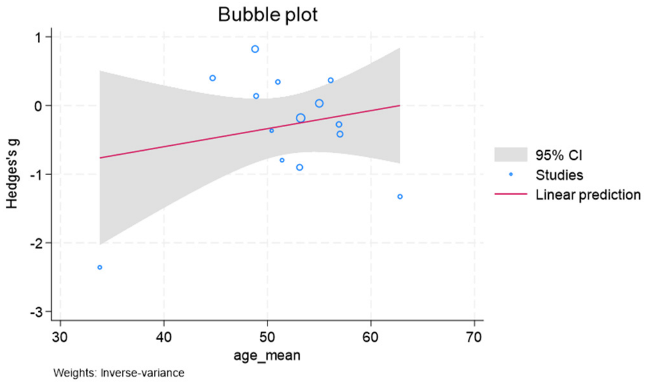

Depression

```
Random-effects meta-regression                     Number of obs  =       14
Method: REML                                 Residual heterogeneity:
                                               tau2 =   .3072
                                             I2 (%) =   79.29
                                                H2 =    4.83
                                        R-squared (%) =   10.44
                                       Wald chi2(1)   =      3.26
                                       Prob > chi2    =    0.0711
------------------------------------------------------------------------------
    _meta_es | Coefficient  Std. err.      z    P>|z|     [95% conf. interval]
-------------+----------------------------------------------------------------
    age_mean |  .0503248   .0278833     1.80   0.071    -.0043255    .1049752
       _cons | -3.053521    1.45986    -2.09   0.036    -5.914794   -.1922492
------------------------------------------------------------------------------
Test of residual homogeneity: Q_res = chi2(12) = 48.13   Prob > Q_res = 0.0000

. estat bubble
```

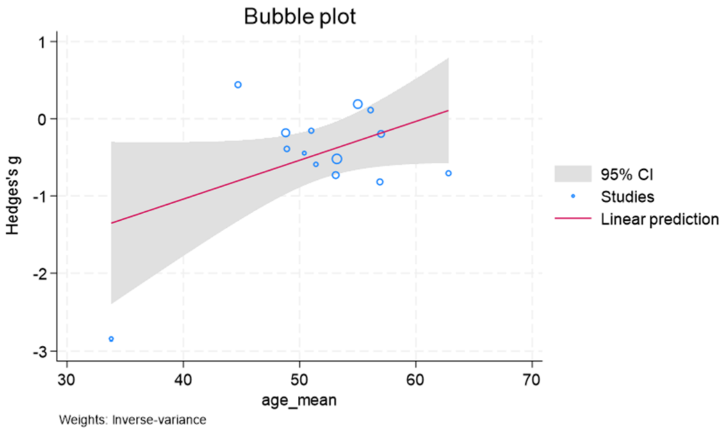

Fatigue

```
Random-effects meta-regression                     Number of obs  =       28
Method: REML                                 Residual heterogeneity:
                                               tau2 =   .5485
                                             I2 (%) =   88.10
                                                H2 =    8.41
                                        R-squared (%) =    3.27
                                       Wald chi2(1)   =      1.73
                                       Prob > chi2    =    0.1879
------------------------------------------------------------------------------
    _meta_es | Coefficient  Std. err.      z    P>|z|     [95% conf. interval]
-------------+----------------------------------------------------------------
    age_mean | -.0333664    .025339    -1.32   0.188      -.08303    .0162972
       _cons |  1.286466   1.350272     0.95   0.341    -1.360018     3.93295
------------------------------------------------------------------------------
Test of residual homogeneity: Q_res = chi2(26) = 200.09  Prob > Q_res = 0.0000

. estat bubble
```

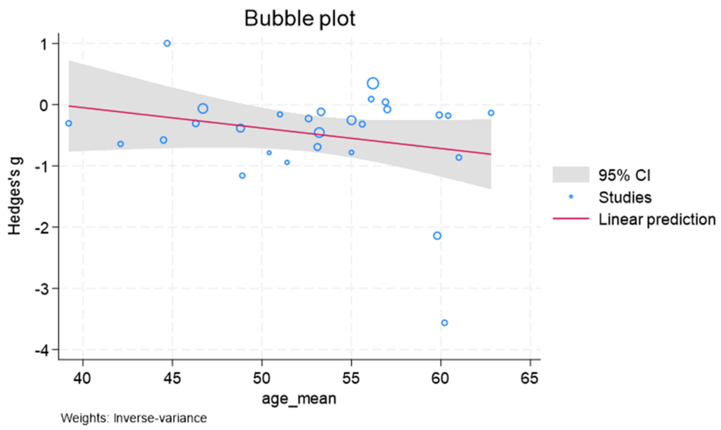

Pain

```
Random-effects meta-regression                    Number of obs  =       13
Method: REML                              Residual heterogeneity:
                                                 tau2 =  .1778
                                               I2 (%) =  70.76
                                                   H2 =   3.42
                                        R-squared (%) =  35.15
                                        Wald chi2(1)  =    5.53
                                        Prob > chi2   =  0.0187
------------------------------------------------------------------------------
    _meta_es | Coefficient  Std. err.      z    P>|z|     [95% conf. interval]
-------------+----------------------------------------------------------------
    age_mean |  -.0472089   .0200757    -2.35   0.019    -.0865566   -.0078613
       _cons |    1.97471   1.040894     1.90   0.058    -.0654044    4.014825
------------------------------------------------------------------------------
Test of residual homogeneity: Q_res = chi2(11) = 37.56   Prob > Q_res = 0.0001

. estat bubble
```

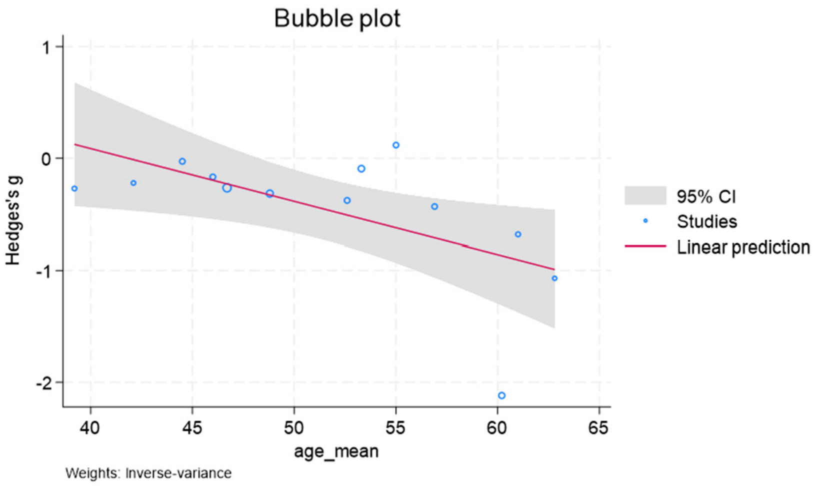

Supplement: Supplementary file 1 [file cancers-16-02962-s001.zip › Figure S20. Meta-regression analyses on exercise benefits related to age for secondary outcomes.pdf]
