# Supplementary material for: Limited Evidence for the Benefits of Exercise in Older Adults with Hematological Malignancies: A Systematic Review and Meta-Analysis
Source: Cancers (Basel). 2024 Aug 25;16(17):2962. doi: 10.3390/cancers16172962 (PMC11393877; doi:10.3390/cancers16172962)

Figure S3. Updated search strategy on March 27, 2024.

**Database**	**Number of documents**
MEDLINE	256
EMBASE	544
CINAHL	261
CENTRAL	407
SCIE/SSCI	336
**Total**	**1804** (number included in PRISMA flowchart Figure 1)
*Imported to Covidence after initial removal of duplicates	1464

Search terms in five databases, 2020 – Update March 27, 2024

Medical Literature Analysis and Retrieval System Online (MEDLINE) (2020–10-20)

#1 Myeloma*[tiab] OR Haematologic cancer*[tiab] OR Hematologic cancer*[tiab] OR Haematological cancer*[tiab] OR Hematological cancer*[tiab] OR Haematologic malignanc*[tiab] OR Hematologic malignanc*[tiab] OR Haematological malignanc*[tiab] OR Hematological malignanc*[tiab]
OR Hematologic Neoplasms[mh] OR Haematologic neoplasm*[tiab] OR Hematologic neoplasm*[tiab] OR Haematological neoplasm*[tiab] OR Hematological neoplasm*[tiab] OR Leukaemia*[tiab] OR Leukemia[mh] OR Leukemia*[tiab] OR Lymphoma[mh] OR Lymphoma*[tiab]

#2 "Cardiovascular training"[tiab] OR Sport*[tiab] OR Sports[mh] OR Crossfit[tiab] OR Dance[tiab] OR Dancing[mh] OR "Endurance training"[tiab] OR Exercise Movement Techniques[mh] OR Exercise[tiab] OR Exercise[mh] OR Exercise Therapy[mh] OR Fitness[tiab] OR Jogg*[tiab] OR Kinesiotherap*[tiab] OR Martial art*[tiab] OR Martial Arts[mh] OR "Muscle strengthening"[tiab] OR "Muscle training"[tiab] OR "Strength training"[tiab] OR "Resistance training"[tiab] OR "Weight lifting"[tiab] OR Physical activit*[tiab] OR Pilates[tiab] OR Yoga[tiab] OR Running[tiab] OR Step activit*[tiab] OR "Tai chi"[tiab] OR "Tai ji"[tiab] OR Walk*[tiab] OR "High-intensity interval training"[tiab] OR "Physical Therapy Modalities"[mh] OR Cardiorespiratory Fitness[mh] OR Circuit-Based Exercise[mh] OR Physical Fitness[mh] OR Resistance Training[mh] OR Walking[mh] OR aerobic*[tiab] OR "circuit-based exercise"[tiab] OR "circuit-based training"[tiab] OR "circuit training"[tiab] OR gymnastics[tiab] OR "high-intensity training"[tiab] OR hydrotherapy[tiab] OR "interval training"[tiab] OR movement therap*[tiab] OR muscle stretch*[tiab] OR swimming[tiab] OR stair climb*[tiab] OR "water therapy"[tiab] OR "weight training"[tiab] OR "outdoor training"[tiab]

#3 (Randomized controlled trial[pt] OR Controlled clinical trial[pt] OR Randomized[tiab] OR Placebo[tiab] OR Randomly[tiab] OR Trial[tiab] OR Groups[tiab]) NOT (Animals [mh] NOT Humans[mh])

#4 RCT[ti] OR RCT[ot]

#5 Cross-Over Studies[mh] OR "Cross-over design"[tiab] OR "Cross-over procedure"[tiab] OR Cross-over study[tiab] OR "Cross-over trial"[tiab] OR "Crossover design"[tiab] OR "Crossover procedure"[tiab] OR Crossover study[tiab] OR "Crossover trial"[tiab]

#6 Pilot Projects[mh] OR "Pilot project"[tiab] OR "Pilot study"[tiab]

#7 Feasibility Studies[mh] OR "Feasibility study"[tiab]

#8
#1 AND #2 AND (#3 OR #4 OR #5 OR #6 OR #7) = 460 [20-10-2020]

Update 27-03-2024

#1
#1 AND #2 AND (#3 OR #4 OR #5 OR #6 OR #7) = 688

#2
#1 AND ("2020/10/01"[CRDT] : "3000"[CRDT] OR "2020/10/01"[EDAT] : "3000"[EDAT] OR "2020/10/01"[MHDA] : "3000"[MHDA]) = **256**

Excerpta Medica Database (EMBASE) (2020-10-20)

1 Myeloma*.ti,ab,kw. OR exp Myeloma/ OR Haematologic cancer*.ti,ab,kw. OR Hematologic cancer*.ti,ab,kw. OR Haematological cancer*.ti,ab,kw. OR Hematological cancer*.ti,ab,kw. OR Haematologic malignanc*.ti,ab,kw. OR Hematologic malignanc*.ti,ab,kw. OR exp Hematologic malignancy/ OR Haematologic neoplasm*.ti,ab,kw. OR Hematologic neoplasm*.ti,ab,kw. OR Haematological neoplasm*.ti,ab,kw. OR Hematological neoplasm*.ti,ab,kw. OR Leukaemia*.ti,ab,kw. OR Leukemia*.ti,ab,kw. OR exp Leukemia/ OR Lymphoma*.ti,ab,kw. OR exp Lymphoma/

2 Cardiovascular training.ti,ab,kw. OR Sport*.ti,ab,kw. OR exp Sport/ OR Crossfit.ti,ab,kw. OR Danc*.ti,ab,kw. OR Endurance training.ti,ab,kw. OR Exercise movement technique*.ti,ab,kw. OR exp Exercise/ OR Exercise.ti,ab,kw. OR Fitness.ti,ab,kw. OR Jogg*.ti,ab,kw. OR exp Kinesiotherapy/ OR Kinesiotherap*.ti,ab,kw. OR Martial art*.ti,ab,kw. OR exp Martial arts/ OR Muscle strengthening.ti,ab,kw. OR Muscle training.ti,ab,kw. OR Strength training.ti,ab,kw. OR Resistance training.ti,ab,kw. OR Weight lifting.ti,ab,kw. OR Physical activit*.ti,ab,kw. OR exp Physical activity/ OR Pilates.ti,ab,kw. OR Yoga.ti,ab,kw. OR Running.ti,ab,kw. OR Step activit*.ti,ab,kw. OR Tai chi.ti,ab,kw. OR Tai ji.ti,ab,kw. OR Walk*.ti,ab,kw. OR Interval training.ti,ab,kw. OR Physical Therapy Modalities.ti,ab,kw. OR Cardiorespiratory fitness/ OR Circuit training/ OR Walking/ OR Stair climbing/ OR aerobic*.ti,ab,kw. OR (circuit ADJ1 exercise).ti,ab,kw. OR (circuit ADJ1 training).ti,ab,kw. OR gymnastics.ti,ab,kw. OR high-intensity training.ti,ab,kw. OR hydrotherapy.ti,ab,kw. OR movement therap*.ti,ab,kw. OR muscle stretch*.ti,ab,kw. OR swimming.ti,ab,kw. OR stair climb*.ti,ab,kw. OR water therapy.ti,ab,kw. OR weight training.ti,ab,kw. OR outdoor training.ti,ab,kw.

3 crossover procedure/ OR double-blind procedure/ OR randomized controlled trial/ OR single-blind procedure/ OR (random* OR factorial* OR crossover* OR cross ADJ1 over* OR placebo* OR doubl* ADJ1 blind* OR singl* ADJ1 blind* OR assign* OR allocat* OR volunteer*).sh,ab,ti.

4 random*.ab,ti. OR (clinical ADJ1 trial*).sh,ab,ti. OR exp health care quality/

5 RCT.ti,kw.

6 (Cross-over ADJ1 design).ti,ab,kw. OR (Cross-over ADJ1 study).ti,ab,kw. OR (Cross-over ADJ1 trial).ti,ab,kw. OR (Crossover ADJ1 design).ti,ab,kw. OR (Crossover ADJ1 study).ti,ab,kw. OR (Crossover ADJ1 trial).ti,ab,kw.

7 Pilot study/ OR Pilot project.ti,ab,kw. OR Pilot study.ti,ab,kw.

8 Feasibility study/ OR Feasibility study.ti,ab,kw.

9 article.pt. OR article in press.pt.

10 1 AND 2 AND (3 OR 4 OR 5 OR 6 OR 7 OR 8) AND 9 = 865

Update 27-03-2024

1 1 AND 2 AND (3 OR 4 OR 5 OR 6 OR 7 OR 8) AND 9 = 1372

2 limit 1 to dc=20201001-20240331 = **544**

Cumulative Index to Nursing and Allied Health Literature (CINAHL) (2020-10-20)

S1 TI Myeloma* OR AB Myeloma* OR TI Haematologic* cancer* OR AB Haematologic* cancer* OR TI Hematologic* cancer* OR AB Hematologic* cancer* OR TI Haematologic* malignanc* AB Haematologic* malignanc* OR TI Hematologic* malignanc* OR AB Hematologic* malignanc* OR MH Hematologic Neoplasms+ OR TI Haematologic* neoplasm* OR AB Haematologic* neoplasm* OR TI Hematologic neoplasm* OR AB Hematologic* neoplasm* OR TI Leukaemia* OR AB Leukaemia* OR MH Leukemia+ OR TI Leukemia* OR AB Leukemia* OR MH Lymphoma+ OR TI Lymphoma* OR AB Lymphoma*

S2 TI Cardiovascular training OR AB Cardiovascular training OR MH Sports+ OR TI Sport* OR AB Sport* OR TI Crossfit OR AB Crossfit OR TI Danc* OR AB Danc* OR MH Dancing+ OR TI Endurance training OR AB Endurance training OR MH Exercise+ OR TI Exercise OR AB Exercise OR MH Therapeutic Exercise+ OR TI Fitness AB Fitness OR TI Jogg* OR AB Jogg* OR TI Kinesiotherap* OR AB Kinesiotherap* OR MH Martial arts OR TI Martial Art* AB Martial Art* OR TI Muscle strengthening OR AB Muscle strengthening OR TI Muscle training OR AB Muscle training OR TI Strength training OR AB Strength training OR TI Resistance training OR AB Resistance training OR TI Weight lifting OR AB Weight lifting OR MH Physical activity OR TI Physical activit* OR AB Physical activit* OR TI Pilates OR AB Pilates OR MH Yoga OR TI Yoga OR AB Yoga OR TI Running OR AB Running OR TI Step activit* OR AB Step activit* OR TI Tai chi OR AB Tai chi OR TI Tai ji OR AB Tai ji OR MH Walking+ OR TI Walk* OR AB Walk* OR TI High-intensity interval training OR AB High-intensity interval training OR MH Physical Therapy OR TI Physical Therapy OR AB Physical Therapy OR MH Physical Fitness OR MH Stair Climbing OR TI aerobic* OR AB aerobic* OR TI circuit W1 exercise OR AB circuit W1 exercise OR TI circuit W1 training OR AB circuit W1 training OR TI gymnastics OR AB gymnastics OR TI high-intensity training OR AB high-intensity training OR TI hydrotherapy OR AB hydrotherapy OR TI interval training OR AB interval training OR TI movement therap* OR AB movement therap* OR TI muscle stretch* OR AB muscle stretch OR TI swimming OR AB swimming OR TI stair climb*[tiab] OR AB stair climb* OR TI water therapy OR AB water therapy OR TI weight training OR AB weight training OR TI outdoor training OR AB outdoor training

S3 S1 AND S2 = 778

Update 27-03-2024

S1 S1 AND S2 = 966

S2 S1 AND (ZD (202010* OR 202011* OR 202012* OR 2021* OR 2022* OR 2023* OR 2024* OR "in process")) = **261**

Cochrane Central Register of Controlled Trials (CENTRAL) (2020-10-20)

#1 Myeloma*:ti,ab,kw OR Haematologic cancer*:ti,ab,kw OR Hematologic cancer*:ti,ab,kw OR Haematological cancer*:ti,ab,kw OR Hematological cancer*:ti,ab,kw OR Haematologic malignanc*:ti,ab,kw OR Hematologic malignanc*:ti,ab,kw OR Haematological malignanc*:ti,ab,kw OR Hematological malignanc*:ti,ab,kw OR mh "Hematologic Neoplasms" OR Haematologic neoplasm*:ti,ab,kw OR Hematologic neoplasm*:ti,ab,kw OR Haematological neoplasm*:ti,ab,kw OR Hematological neoplasm*:ti,ab,kw OR Leukaemia*:ti,ab,kw OR mh "Leukemia" OR Leukemia*:ti,ab,kw OR mh "Lymphoma" OR Lymphoma*:ti,ab,kw

#2 "Cardiovascular training":ti,ab,kw OR Sport*:ti,ab,kw OR mh "Sports" OR Crossfit:ti,ab,kw OR Danc*:ti,ab,kw OR mh "Dancing" OR "Endurance training":ti,ab,kw OR mh "Exercise Movement Techniques" OR Exercise:ti,ab,kw OR mh "Exercise" OR mh "Exercise Therapy" OR Fitness:ti,ab,kw OR Jogg*:ti,ab,kw OR Kinesiotherap*:ti,ab,kw

OR Martial art*:ti,ab,kw OR mh "Martial Arts" OR "Muscle strengthening":ti,ab,kw OR "Muscle training":ti,ab,kw OR "Strength training":ti,ab,kw OR "Resistance training":ti,ab,kw OR "Weight lifting":ti,ab,kw OR Physical activit*:ti,ab,kw OR Pilates:ti,ab,kw OR Yoga:ti,ab,kw OR Running:ti,ab,kw OR Step activit*:ti,ab,kw OR "Tai chi":ti,ab,kw OR "Tai ji":ti,ab,kw OR Walk*:ti,ab,kw OR "High-intensity interval training":ti,ab,kw OR mh "Physical Therapy Modalities" OR mh "Cardiorespiratory Fitness" OR mh "Circuit-Based Exercise" OR mh "Physical Fitness" OR mh "Resistance Training" OR mh "Walking" OR aerobic*:ti,ab,kw OR "circuit-based exercise":ti,ab,kw OR "circuit-based training":ti,ab,kw OR "circuit training":ti,ab,kw OR gymnastics:ti,ab,kw OR "high-intensity training":ti,ab,kw OR "hydrotherapy":ti,ab,kw OR "interval training":ti,ab,kw OR movement therap*:ti,ab,kw OR muscle stretch*:ti,ab,kw OR "swimming":ti,ab,kw OR stair climb*:ti,ab,kw OR "water therapy":ti,ab,kw OR "weight training":ti,ab,kw OR "outdoor training":ti,ab,kw

#3 #1 AND #2 = 801

Update 27-03-2024

#1 #1 AND #2 = 1129

with Cochrane Library publication date from Oct 2020 to Apr 2024 = **407**

Science Citation Index Expanded / Social Sciences Citation Index (SCIE/SSCI) (2020-10-20)

#1 TS=(Myeloma* OR "Haematologic cancer*" OR "Hematologic cancer*" OR "Haematological cancer*" OR "Hematological cancer*" OR "Haematologic malignanc*" OR "Hematologic malignanc*" OR "Haematologic neoplasm*" OR "Hematologic neoplasm*" OR "Haematological neoplasm*" OR "Hematological neoplasm*" OR Leukaemia* OR Leukemia* OR Lymphoma*)

#2 TS=("Cardiovascular training" OR Sport OR Crossfit OR Danc* OR "Endurance training" OR "Exercise movement technique*" OR Exercise OR Fitness OR Jogg* OR Kinesiotherap* OR "Martial art*" OR "Muscle strengthening" OR "Muscle training" OR "Strength training" OR "Resistance training" OR "Weight lifting" OR "Physical activit*" OR Pilates OR Yoga OR Running OR "Step activit*" OR "Tai chi" OR Tai ji OR Walk* OR "Interval training" OR "Physical Therap*" OR aerobic* OR (circuit NEAR/1 exercise) OR (circuit NEAR/1 training) OR gymnastics OR "high-intensity training" OR hydrotherapy OR "movement therap*" OR "muscle stretch*" OR swimming OR "stair climb*" OR "water therapy" OR "weight training" OR "outdoor training")

#3 TS= clinical trial* OR TS=research design OR TS=comparative stud* OR TS=evaluation stud* OR TS=controlled trial* OR TS=follow-up stud* OR TS=prospective stud* OR TS=random* OR TS=placebo* OR TS=(single blind*) OR TS=(double blind*)

#4 TS=(RCT)

#5 TS= ((Cross-over NEAR/1 design) OR (Cross-over NEAR/1 study) OR (Cross-over NEAR/1 trial) OR (Crossover NEAR/1 design) OR (Crossover NEAR/1 study) OR (Crossover NEAR/1 trial))

#6 TS=("Pilot project" OR "Pilot study")

#7 TS=("Feasibility study")

#8 #1 AND #2 AND (#3 OR #4 OR #5 OR #6 OR #7) AND DOCUMENT TYPES: (Article) = 842

Update 27-03-2024

#1 #1 AND #2 AND (#3 OR #4 OR #5 OR #6 OR #7) AND DOCUMENT TYPES: (Article) =

#2 #1 AND LD=(2020-10-01/2024-03-31) = **336**

Covidence Flowchart: Of the total hits (n= 1804) and after initial removal of duplicates, 1464 studies were imported into Covidence for further screening of duplicates and for relevance.

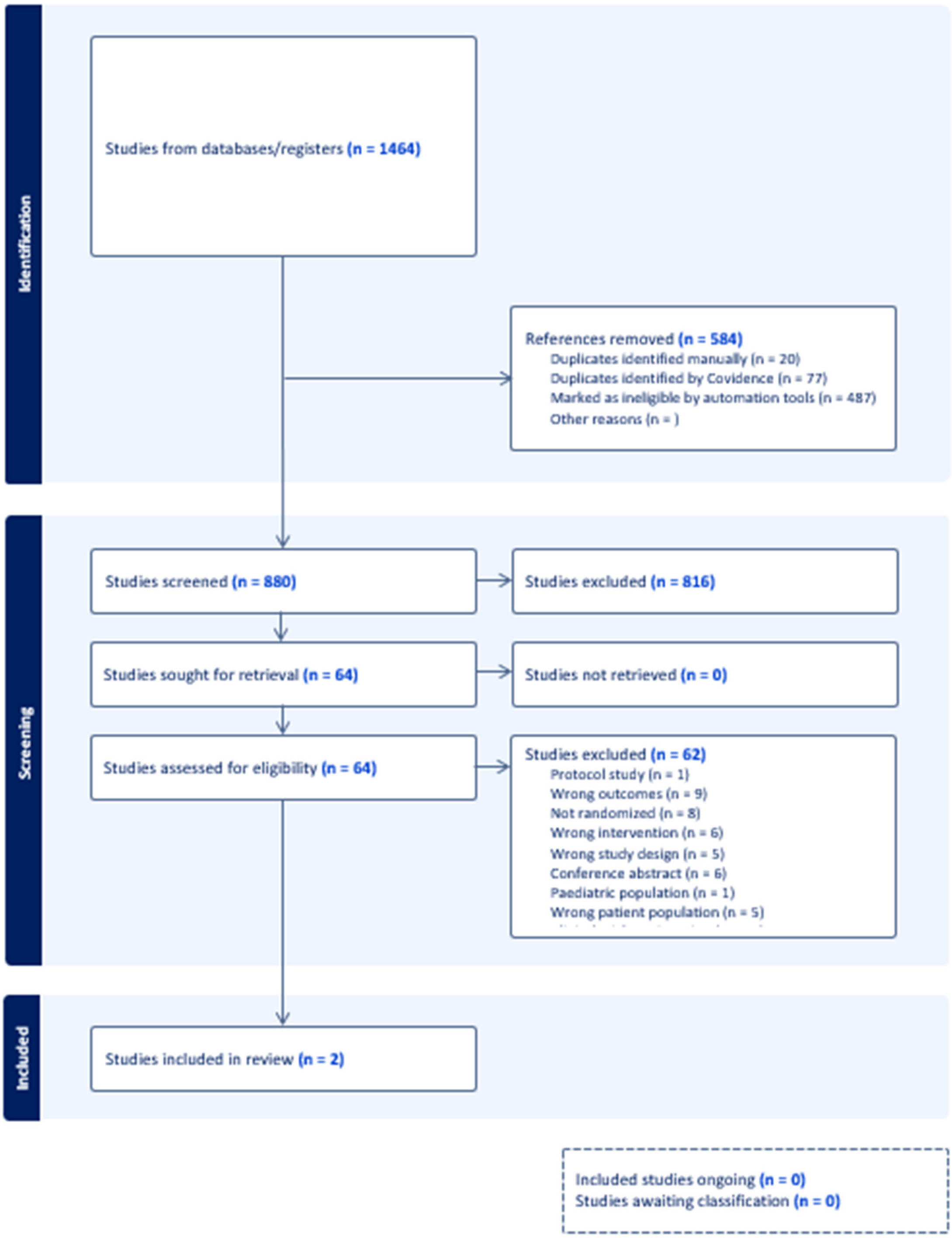

Supplement: Supplementary file 1 [file cancers-16-02962-s001.zip › Figure S3. Search strategy and PRISMA flow diagram for study selection (March 2024).pdf]
