# Supplementary material for: Limited Evidence for the Benefits of Exercise in Older Adults with Hematological Malignancies: A Systematic Review and Meta-Analysis
Source: Cancers (Basel). 2024 Aug 25;16(17):2962. doi: 10.3390/cancers16172962 (PMC11393877; doi:10.3390/cancers16172962)

Figure S6. Meta-regression analyses on exercise benefits related to age for primary outcomes, physical function and QoL global

Physical function

```
Random-effects meta-regression                    Number of obs  =       21
Method: REML                                Residual heterogeneity:
                                          tau2 =  .08778
                                         I2 (%) =   51.61
                                            H2 =    2.07
                                     R-squared (%) =   18.16
                                   Wald chi2(1)   =      7.73
                                   Prob > chi2    =     0.0054
------------------------------------------------------------------------------
   _meta_es | Coefficient  Std. err.      z    P>|z|     [95% conf. interval]
-------------+----------------------------------------------------------------
   age_mean |  -.0400944     .01442    -2.78   0.005     -.068357   -.0118317
      _cons |   2.395674   .7575421     3.16   0.002     .9109188    3.880429
------------------------------------------------------------------------------
Test of residual homogeneity: Q_res = chi2(19) = 42.60   Prob > Q_res = 0.0015

. estat bubble
```

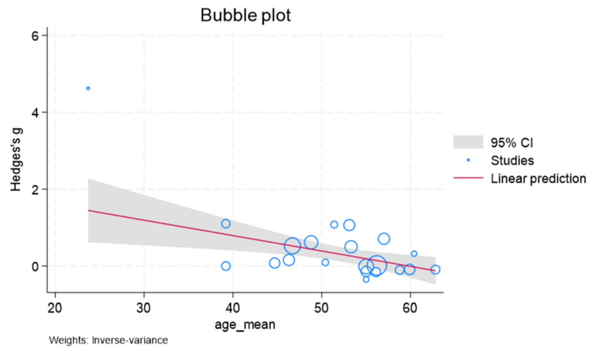

QoL global

```
Random-effects meta-regression                    Number of obs  =       27
Method: REML                                Residual heterogeneity:
                                          tau2 =  .6109
                                         I2 (%) =   88.31
                                            H2 =    8.56
                                     R-squared (%) =    2.15
                                   Wald chi2(1)   =      1.57
                                   Prob > chi2    =     0.2104
------------------------------------------------------------------------------
   _meta_es | Coefficient  Std. err.      z    P>|z|     [95% conf. interval]
-------------+----------------------------------------------------------------
   age_mean |   .0246995   .0197211     1.25   0.210    -.0139532    .0633522
      _cons |  -.9091129   1.032109    -0.88   0.378    -2.932009    1.113783
------------------------------------------------------------------------------
Test of residual homogeneity: Q_res = chi2(25) = 154.92   Prob > Q_res = 0.0000

. estat bubble
```

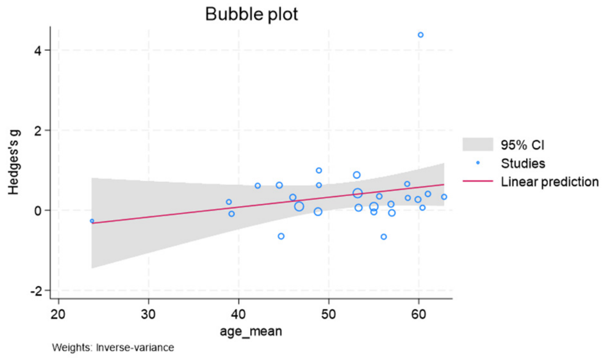

Supplement: Supplementary file 1 [file cancers-16-02962-s001.zip › Figure S6. Meta-regression analyses on exercise benefits related to age for primary outcomes, physical function and QoL global.pdf]
