# Supplementary material for: Limited Evidence for the Benefits of Exercise in Older Adults with Hematological Malignancies: A Systematic Review and Meta-Analysis
Source: Cancers (Basel). 2024 Aug 25;16(17):2962. doi: 10.3390/cancers16172962 (PMC11393877; doi:10.3390/cancers16172962)

Figure S7. Subgroup analyses on primary outcome; physical function.

Physical function	Number of studies	Effect size (Hedges's g) with 95% CI	p-value
**Age of participants**			
Below 60 years	22	0.32 [0.14, 0.50]	0.001
Above 60 years	3	-0.01 [-0.38, 0.37]	0.978
Test of group differences: $Q_b(1) = 2.31$, $p = 0.13$			
**Diagnosis**			
Acute leukemia	6	0.94 [0.02, 1.87]	0.046
Multiple myeloma	4	0.01 [-0.23, 0.25]	0.944
Mixed diagnoses	15	0.24 [0.07, 0.42]	0.007
Test of group differences: $Q_b(2) = 5.11$, $p = 0.08$			
**Antineoplastic treatment**			
Chemotherapy	9	0.51 [0.02, 1.01]	0.043
Allogeneic HSCT	4	0.54 [0.14, 0.95]	0.009
Autologous HSCT	3	-0.00 [-0.33, 0.33]	0.995
Autologous HSCT and Allogeneic HSCT	5	0.29 [0.00, 0.58]	0.048
Different treatments	4	0.01 [-0.21, 0.24]	0.918
Test of group differences: $Q_b(4) = 8.65$, $p = 0.07$			
**Type of exercise**			
Aerobic exercise	4	0.31 [-0.18, 0.79]	0.214
Strength exercise	3	0.20 [-0.28, 0.68]	0.412
Combined aerobic and strength exercise	17	0.27 [0.07, 0.46]	0.007
Either aerobic or strength exercise	1	4.62 [2.41, 6.84]	0.000
Test of group differences: $Q_b(3) = 14.89$, $p = 0.00$			
**Timing of exercise**			
Before treatment	1	0.25 [-0.86, 1.37]	0.656
During treatment	10	0.57 [0.13, 1.01]	0.011
After treatment	8	0.10 [-0.10, 0.31]	0.330
Before and/or during and/or after treatment	6	0.32 [0.03, 0.60]	0.028
Test of group differences: $Q_b(3) = 4.14$, $p = 0.25$			
**Delivery mode**			
Supervised	15	0.40 [0.13, 0.66]	0.003
Partly supervised	7	0.18 [-0.09, 0.46]	0.195
Unsupervised	3	0.06 [-0.20, 0.33]	0.633
Test of group differences: $Q_b(2) = 3.12$, $p = 0.21$			
**Individual or groupbased exercise**			
Individual	22	0.26 [0.10, 0.42]	0.001
Group-based	1	-0.14 [-0.71, 0.43]	0.629
Combi of individual and group-based	2	0.87 [0.23, 1.51]	0.008
Test of group differences: $Q_b(2) = 5.34$, $p = 0.07$			

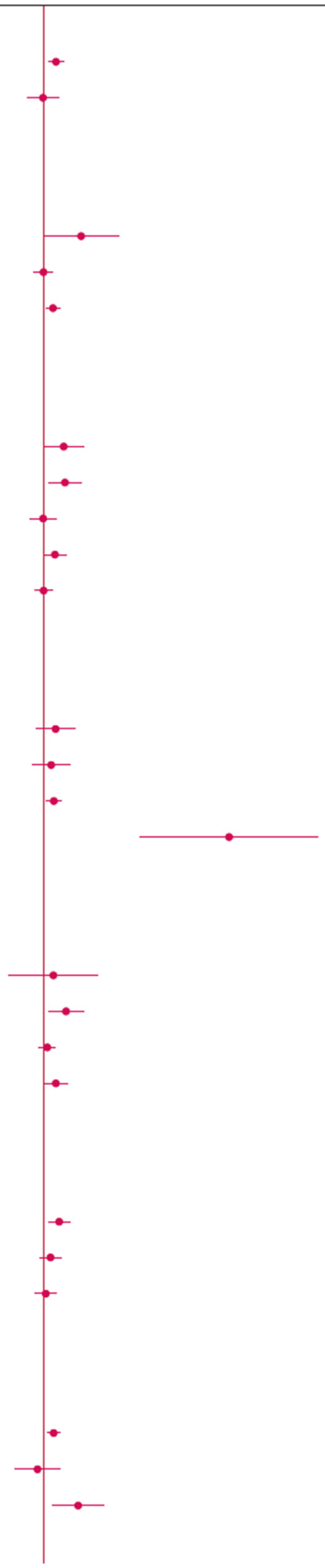

Supplement: Supplementary file 1 [file cancers-16-02962-s001.zip › Figure S7. Subgroup analyses on primary outcome; physical function.pdf]
