# Supplementary material for: Limited Evidence for the Benefits of Exercise in Older Adults with Hematological Malignancies: A Systematic Review and Meta-Analysis
Source: Cancers (Basel). 2024 Aug 25;16(17):2962. doi: 10.3390/cancers16172962 (PMC11393877; doi:10.3390/cancers16172962)
Supplement: Supplementary file 1 [file cancers-16-02962-s001.zip › Table S1. Search strategy on November 13, 2023, implemented in ClinicalTrials.gov.pdf]

Table S1. Search strategy on November 13, 2013, implemented in ClinicalTrial.gov.

**Condition/Disease**	**Intervention**	**Limits**
hematologic; hematology; Haematologic hematologic diseases; Hematological Diseases; Hematologic and Lymphocytic Disorder; Blood Diseases	Exercise<br>Physical activity	Timeframe 01.01.17-13.11.23

Inclusion: Population 60+ years

Exclusion: Studies with mixed population (multiple cancer types) and studies with population 18+

220 hits
