# Supplementary material for: Limited Evidence for the Benefits of Exercise in Older Adults with Hematological Malignancies: A Systematic Review and Meta-Analysis
Source: Cancers (Basel). 2024 Aug 25;16(17):2962. doi: 10.3390/cancers16172962 (PMC11393877; doi:10.3390/cancers16172962)
Supplement: Supplementary file 1 [file cancers-16-02962-s001.zip › Table S2. Outcome measurement tests and instruments chosen for meta-analysis.pdf]

Table S2. Outcome measurement tests and instruments chosen for meta-analysis.

	Objectives				PROMs								
**Author**	**Physical function**	**Aerobic capacity**	**Muscle strength**	**Body compositio n**	**Physical activity**	**QoL Global**	**QoL Emotional**	**QoL Functional**	**QoL Physical**	**Anxiety**	**Depression**	**Fatigue**	**Pain**
**Accogli** et al. 2022, Italy	TUG					EORTC QLQ-C30	NCCN Distress Thermometer	EORTC QLQ-C30				FACIT-F	
**Alibhai** et al. 2014, Canada	6MWT		GRIP Test			EORTC QLQ-C30	EORTC QLQ-C30		EORTC QLQ-C30	HADS	HADS	FACT-F	
**Alibhai** et al. 2015, Canada	6MWT	VO2 Peak - modified	10-time-repeated STS			EORTC QLQ-C30	EORTC QLQ-C30		EORTC QLQ-C30	HADS	HADS	FACT-F	
**Baumann** et al. 2010, Germany		Modified endurance test	Max test, Isomet Knee Ext			EORTC QLQ-C30	EORTC QLQ-C30		EORTC QLQ-C30			EORTC QLQ-C30	EORTC QLQ-C30
**Baumann** et al. 2011, Germany		Modified endurance test	Max test, Isomet Knee Ext	BMI		EORTC QLQ-C30	EORTC QLQ-C30		EORTC QLQ-C30			EORTC QLQ-C30	EORTC QLQ-C30
**Bayram** et al. 2024, Turkey		VO2 Max				EORTC QLQ-C30							
**Bird** et al. 2010, UK	SWT					GLQOL G	SF-36		SF-36				SF-36
**Bryant** et al. 2018, USA	6MWT		Leg strength and grip strength				SF-12		SF-12	PROMIS	PROMIS	PROMIS	
**Chang** et al. 2008, Taiwan	12MWT									POMS	POMS	BFI Worst	
**Chen** et al. 2021, China						CMSAS	CMSAS		CMSAS				
**Chow** et al. 2020, USA	Acceleromet er						PROMIS		PROMIS				
**Chuang** et al. 2017, Taiwan						EORTC QLQ-C30	EORTC QLQ-C30		EORTC QLQ-C30			EORTC QLQ-C30	EORTC QLQ-C30
**Cohen** et al. 2004, USA										STAI	CES-D	BFI	
**Coleman** et al. 2003, USA		Modified Balke	Max test Isoton upper and lower extremity	LBW BodPod			POMS					POMS	
**Coleman** et al. 2012, USA	6MWT											FACT-F	
**Courneya** et al. 2009, Canada		VO2 Max		LM DEXA		FACT-AN	Happiness Scale		FACT-TOI	STAI	CES-D	FACT-An	
**Defor** et al. 2007, USA	KPS												

**Eckert** et al. 2022, USA										PROMIS	PROMIS	PROMIS	PROMIS
**Furzer** et al. 2016, Australia		Aerobic Power Index	Max test, Isokin Knee Ext	LM DEXA		FACT-G		FACT- TOI	SF-36	HADS	HADS	SCFS	
**Gallardo-Rodriquez** et al. 2023, Mexico	6MWT		STS	SMM SECA Bioimpeda nce		FACT-Leu	FACT-Leu	FACT-Leu	FACT-Leu				
**Hacker** et al. 2017, USA	TUG	Timed stair climb	STS		GLTEQ	EORTC QLQ-C30	EORTC QLQ-C30		EORTC QLQ-C30			EORTC QLQ-C30	EORTC QLQ-C30
**Hacker** et al. 2022, USA	TUG		STS			EORTC QLQ-C30	EORTC QLQ-C30		EORTC QLQ-C30	PROMIS	PROMIS	EORTC QLQ-C30	EORTC QLQ-C30
**Hathiramani** et al. 2020, UK						EORTC QLQ-C30	EORTC QLQ-C30		EORTC QLQ-C30			EORTC QLQ-C30	EORTC QLQ-C30
**Huberty** et al. 2019, USA						PROMIS	PROMIS		PROMIS	PROMIS	PROMIS	MPN-SAF	PROMIS
**Hung** et al. 2014, Australia				LBM BodPod	AAS	EORTC QLQ-C30	EORTC QLQ-C30		EORTC QLQ-C30				
**Jacobsen** et al. 2014, USA							SF-36		SF-36				
**Jarden** et al. 2009, Denmark	2MSC	VO2 Max	Max test, Isoton Knee Ext	BMI		EORTC QLQ-C30	EORTC QLQ-C30	FACT-An	EORTC QLQ-C30			EORTC QLQ-C30	EORTC QLQ-C30
**Jarden** et al. 2016, Denmark	6MWT	VO2 Max	STS			EORTC QLQ-C30	EORTC QLQ-C30	FACT-An	FACT-An	HADS	HADS	EORTC QLQ-C30	
**Kim** et al. 2005, South Korea										STAI	BDI		
**Knols** et al. 2011, Switzerland	6MWT		Max test, Isomet Knee Ext	LM DEXA	IPAQ	EORTC QLQ-C30	EORTC QLQ-C30		EORTC QLQ-C30			FACT-AN	EORTC QLQ-C30
**Kobayashi** et al. 2020, Japan	TUG		STS			POMS				POMS	POMS	POMS	
**Koutoukidis** et al. 2020, UK	Acceleromet er	VO2 Peak REL	Max test, Isokin Knee Ext	MM Tanita			FACT-G	FACT-G		HADS	HADS	FACIT-F	
**McCourt** et al. 2023, UK	6MWT		STS			EORTC QLQ-C30		FACT-BMT TOI				FACIT-F	
**Mello** et al., 2003, Brazil			Max test, Isomet Knee Ext DM										
**Oechsle** et al. 2014, Germany		VO2 Max											
**Pahl** et al. 2018, Germany	TUG		CRT power										

**Pahl** et al. 2020, Germany		VO2 Max	Max test, Isokin Knee Ext	FFM BodPod		EORTC QLQ-C30	EORTC QLQ-C30		EORTC QLQ-C30			MFI	
**Persoon** et al., 2017, Nederland	Accelerometer	VO2 Peak	Max test, Isomet Knee Ext	BMI	PASE	EORTC QLQ-C30	EORTC QLQ-C30		EORTC QLQ-C30	HADS	HADS	MFI	
**Potiaumpai** et al. 2021, USA	6MWT					FACT-BMT		FACT-BMT	FACT-BMT				
**Safran** et al. 2022, Turkey	TUG		Max test, Isomet Knee Ext										
**Schumacher** et al**.** 2018, Germany	2MWT		GRIP Test			FACT-BMT		FACT-BMT FWB	FACT-BMT PWB	HADS	HADS		
**Shelton** et al. 2009, USA	6MWT		STS									BFI	
**Streckmann** et al. 2014, Germany		POWER Max			Activity MET	EORTC QLQ-C30							EORTC QLQ-C30
**Vallerand** et al. 2018, Canada					GLTEQ		SF-36		SF-36			FACT-F	SF-36
**Waked** et al. 2019, Egypt				FFM DEXA									
**Wehrle** et al. 2019, Germany		POWER Max	Max test, Isokin Knee Ext			EORTC QLQ-C30	EORTC QLQ-C30		EORTC QLQ-C30				
**Wiskemann** et al. 2011, Germany	6MWT		Max test, Isomet BLE			EORTC QLQ-C30	EORTC QLQ-C30		EORTC QLQ-C30	HADS	HADS	EORTC QLQ-C30	EORTC QLQ-C30
**Wood** et al. 2020, USA	6MWT	VO2 Max Rel											
**Yeh** et al. 2016, Taiwan												11-point rating scale	

2MSC: 2 min stair climb; **12MWT:** 12 min walk test; **6MWT:** 6 min walk test; **AAS:** Active Australia Survey; **Activity MET:** Activity, metabolic equivalent; **BDI:** Beck Depression Inventory; **BFI**: Brief Fatigue Inventory; **BLE**: Bilateral Lower Extremities; **BMI**: Body Mass Index; **CES-D**: Center for Epidemiological Studies Depression Scale; **CMSAS**: Condensed Memorial Symptom Assessment Scale; **CRT**: Chair rising test; **DEXA**: Dual x-ray absorptiometry; **DM**: Dominant; **EORTC QLQ-C30**: The European Organization for Research and Treatment of Cancer Core Quality of Life Questionnaire; **Ext**: Extension; **FACT**: Functional Assessment of Cancer Therapy; **FACT-An**: The Functional Assessment of Cancer Therapy – Anemia; **FACT-BMT**: The Functional Assessment of Cancer Therapy – Bone Marrow Transplantation; **FACIT-F**: The Functional Assessment of Chronic Illness Therapy Fatigue Scale; **FACT-G**: The Functional Assessment of Cancer Therapy – General; **FACT-Leu**: Functional Assessment of Cancer Therapy – Leukemia; **FFM**: Fat Free Mass; **GLTEQ**: Godin Leisure-Time Exercise Questionnaire; **GLQOL**: Graham and Longman Quality of Life Scale; **HADS**: Hospital Anxiety and Depression Scale; **IPAQ**: International Physical Activity Questionnaire; **Isomet:** Isometric; **Isokin**: Isokinetic; **Isoton**: Isotonic; **KPS**: Karnofsky performance score, **LBM**: Lean Body Mass; **LBW**: Lean Body Weight; **LM**: Lean Mass; **MFI**: Multidimensional Fatigue Inventory; **MM**: Muscle mass; **MPN-SAF**: The Myeloproliferative Neoplasm Symptom Assessment Form; **NCCN Distress Thermometer**: National Comprehensive Cancer Network Distress Thermometer; **PASE**: Physical Activity Scale for the Elderly; **POMS**: Profile of Mood States; **PROMIS**: Patient Reported Outcomes Measurement Information System; **Rel**: Relative; **SCFS**: Schwartz Cancer Fatigue Scale; **SF-12**: 12-Item Short Form Survey; **SF-36**: 36-Item Short Form Survey; **SMM**: Skeletal muscle mass; **STAI**: Spielberger State Anxiety Inventory; **STS**: Sit to Stand; **SWT**: Shuttle Walk Test, **TANITA**: Bioelectrical impedance analysis; **TOI**: Trial Outcome Index; **TUG**: Timed up and go; **VO_2 Max Rel**: Volume Oxygen Maximal Relative / Maximal aerobic capacity relative
