# Supplementary figures and images for: Limited Evidence for the Benefits of Exercise in Older Adults with Hematological Malignancies: A Systematic Review and Meta-Analysis
Source: Cancers (Basel). 2024 Aug 25;16(17):2962. doi: 10.3390/cancers16172962 (PMC11393877; doi:10.3390/cancers16172962)

Figure S10. Forest plot depicting the effects of exercise on muscle strength.

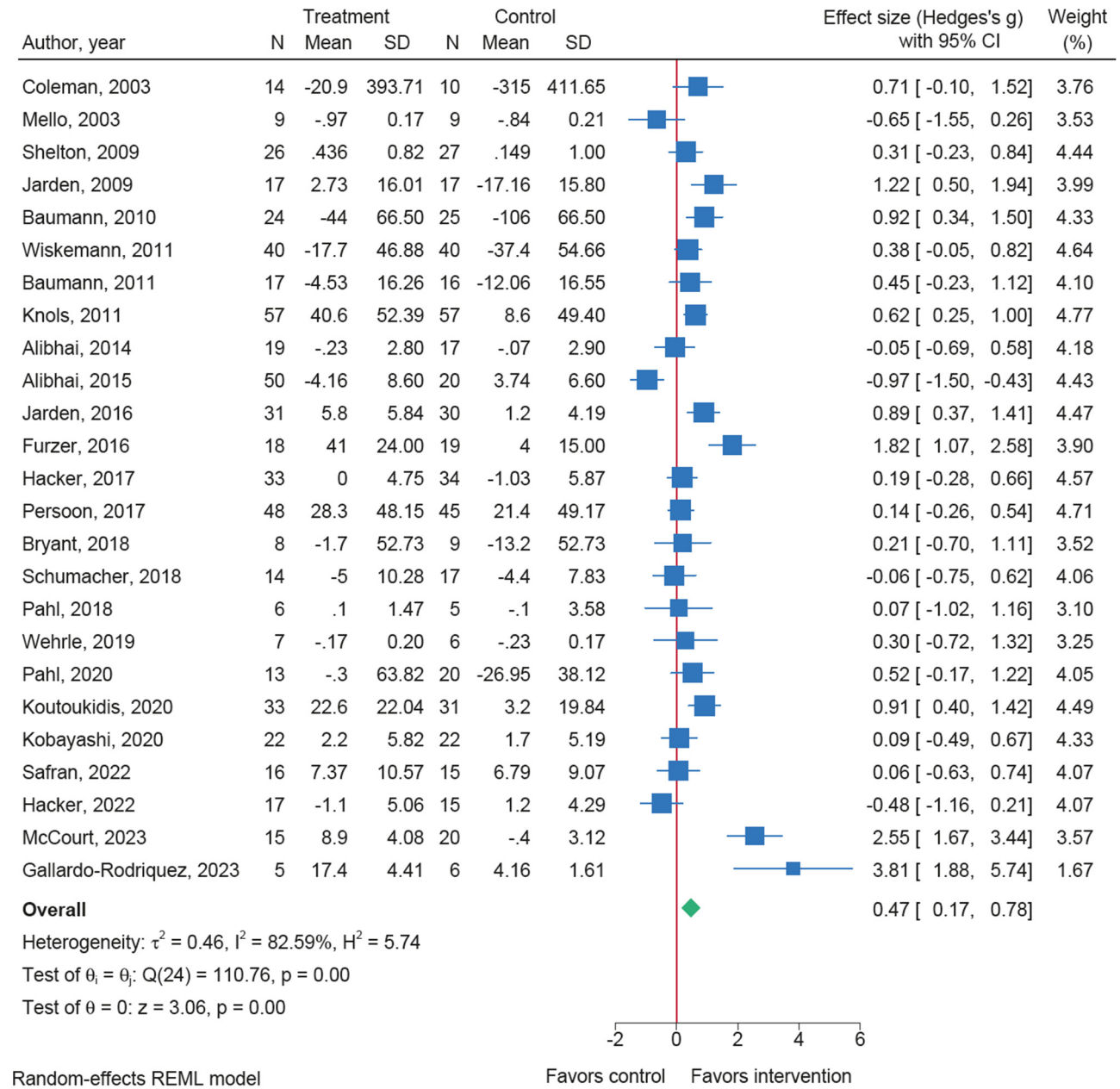

Supplement: Supplementary file 1 [file cancers-16-02962-s001.zip › Figure S10. Forest plot depicting the effects of exercise on muscle strength.pdf]

Figure S11. Forest plot depicting the effects of exercise on body composition.

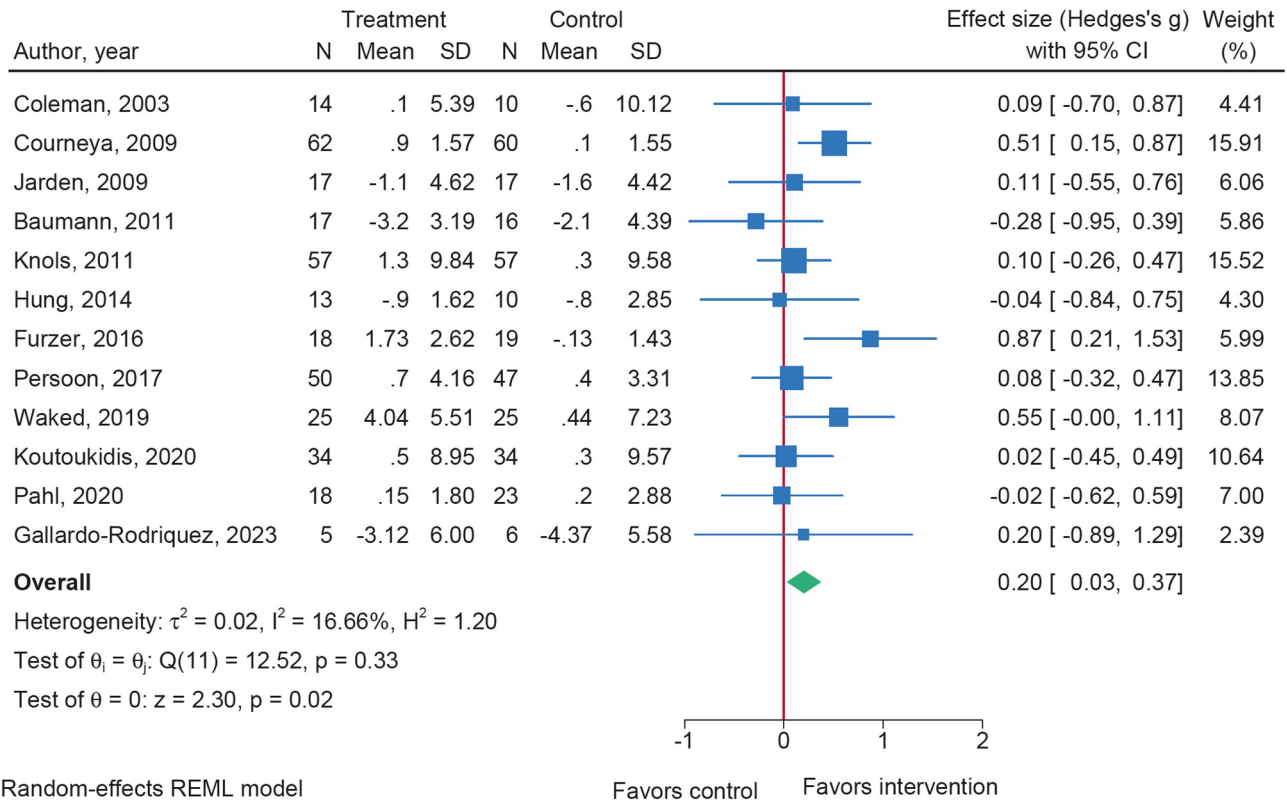

Supplement: Supplementary file 1 [file cancers-16-02962-s001.zip › Figure S11. Forest plot depicting the effects of exercise on body composition.pdf]

Figure S12. Forest plot depicting the effects of exercise on physical activity.

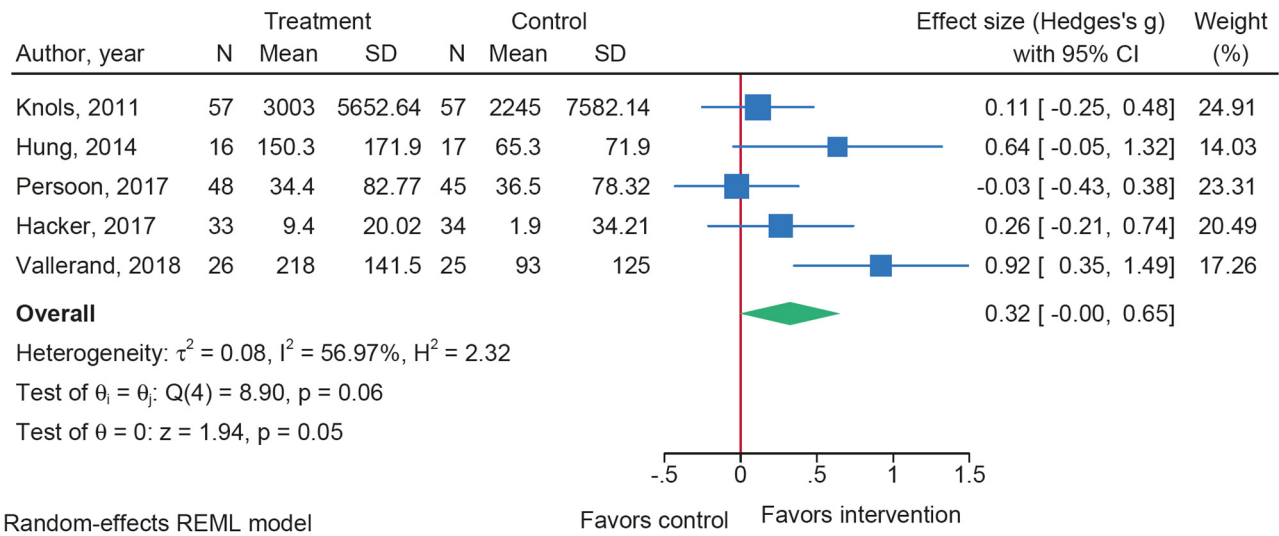

Supplement: Supplementary file 1 [file cancers-16-02962-s001.zip › Figure S12. Forest plot depicting the effects of exercise on physical activity.pdf]

Figure S13. Forest plot depicting the effects of exercise on QoL emotional.

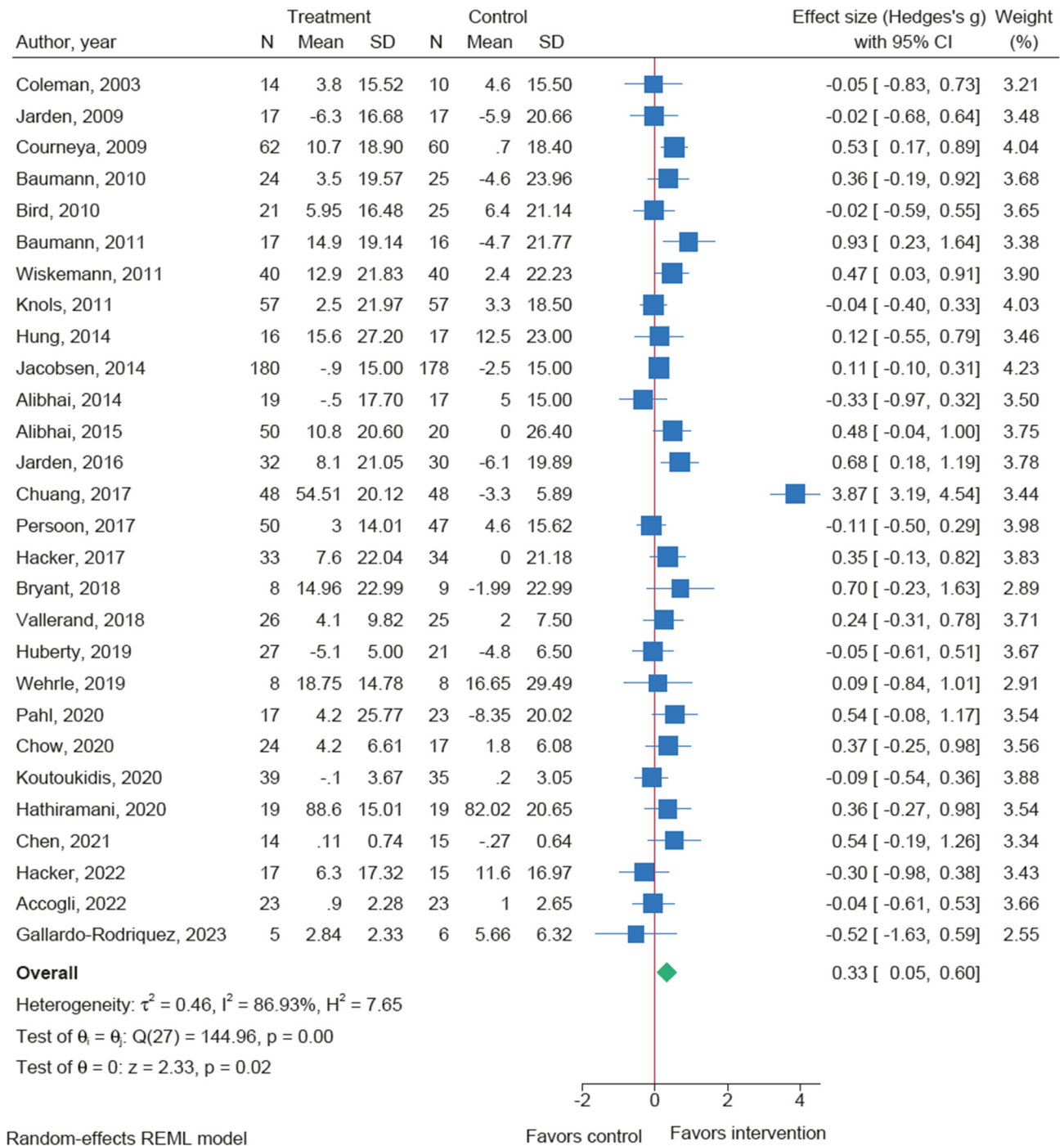

Supplement: Supplementary file 1 [file cancers-16-02962-s001.zip › Figure S13. Forest plot depicting the effects of exercise on QoL emotional.pdf]

Figure S14. Forest plot depicting the effects of exercise on QoL functional.

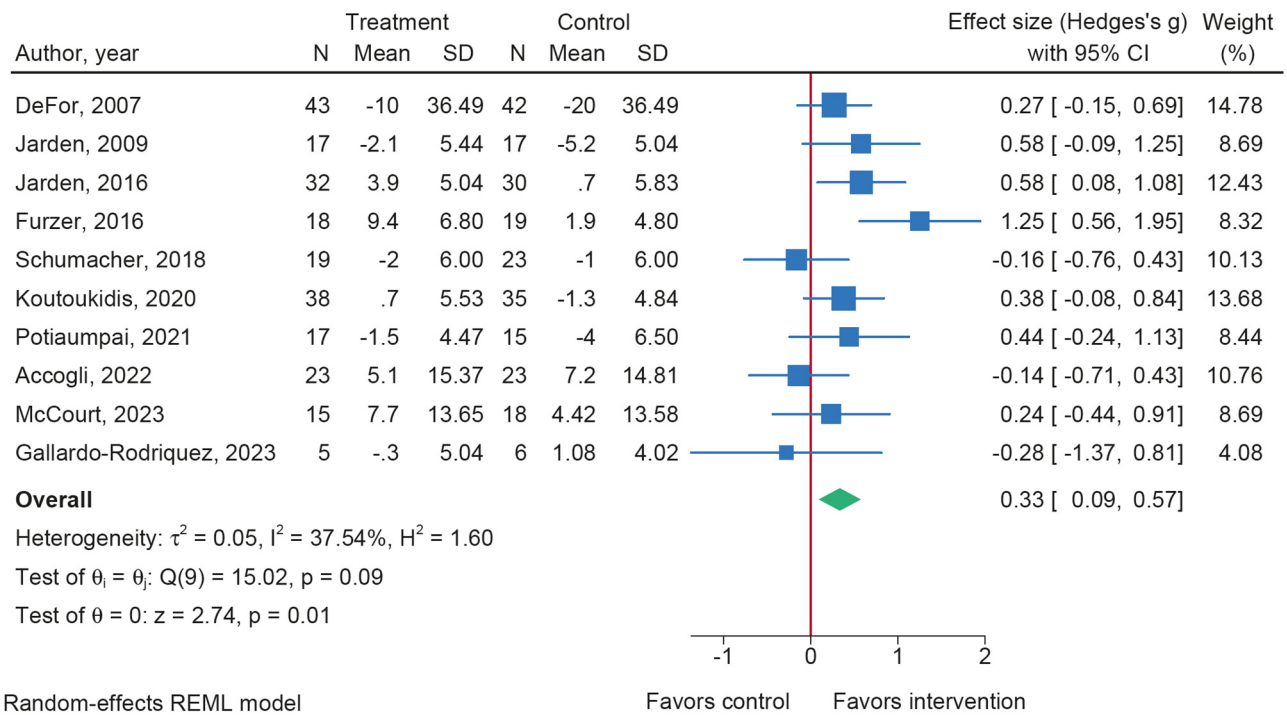

Supplement: Supplementary file 1 [file cancers-16-02962-s001.zip › Figure S14. Forest plot depicting the effects of exercise on QoL functional.pdf]

Figure S15. Forest plot depicting the effects of exercise on QoL physical.

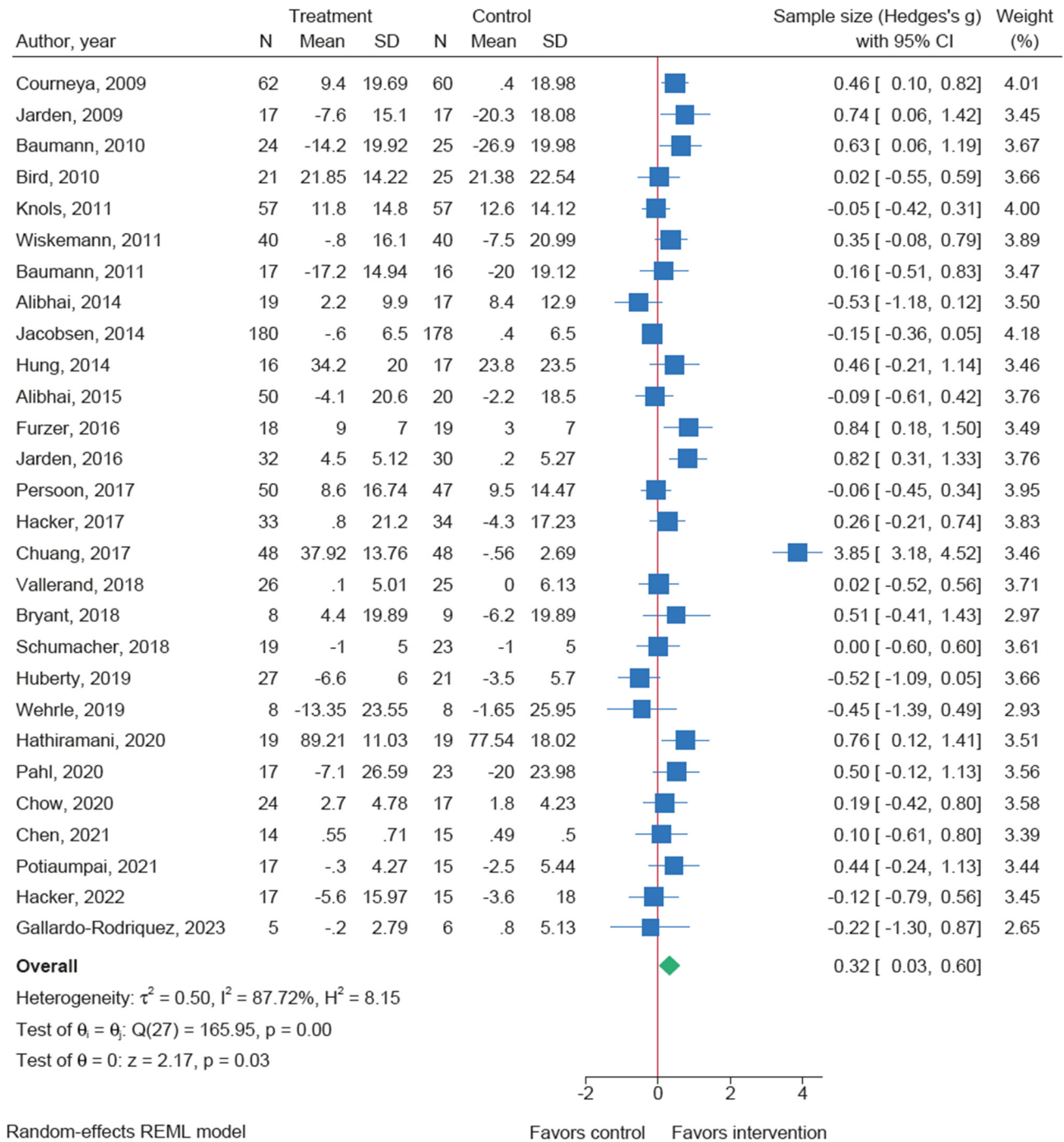

Supplement: Supplementary file 1 [file cancers-16-02962-s001.zip › Figure S15. Forest plot depicting the effects of exercise on QoL physical.pdf]

Figure S16. Forest plot depicting the effects of exercise on anxiety.

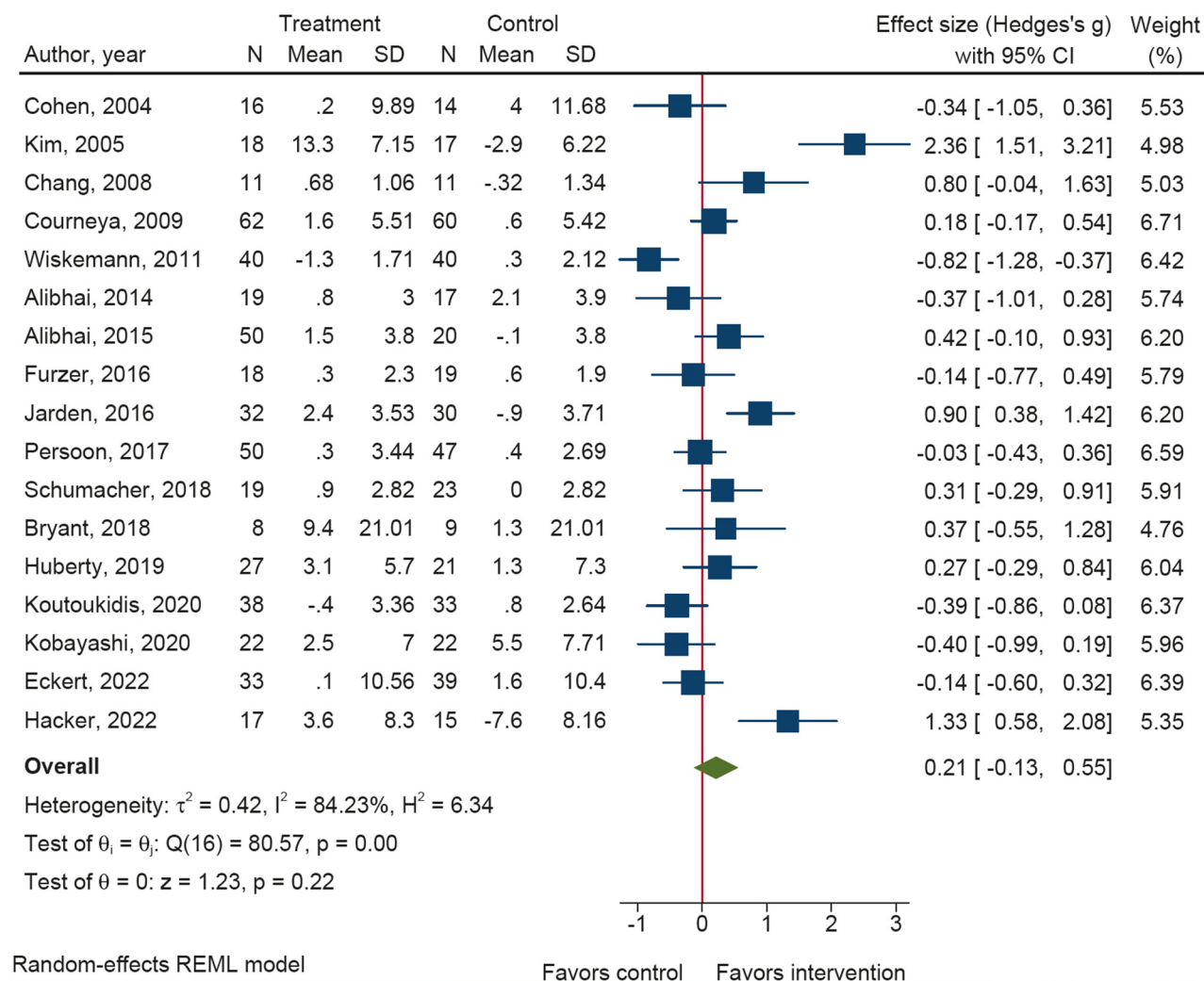

Supplement: Supplementary file 1 [file cancers-16-02962-s001.zip › Figure S16. Forest plot depicting the effects of exercise on anxiety.pdf]

Figure S17. Forest plot depicting the effects of exercise on depression.

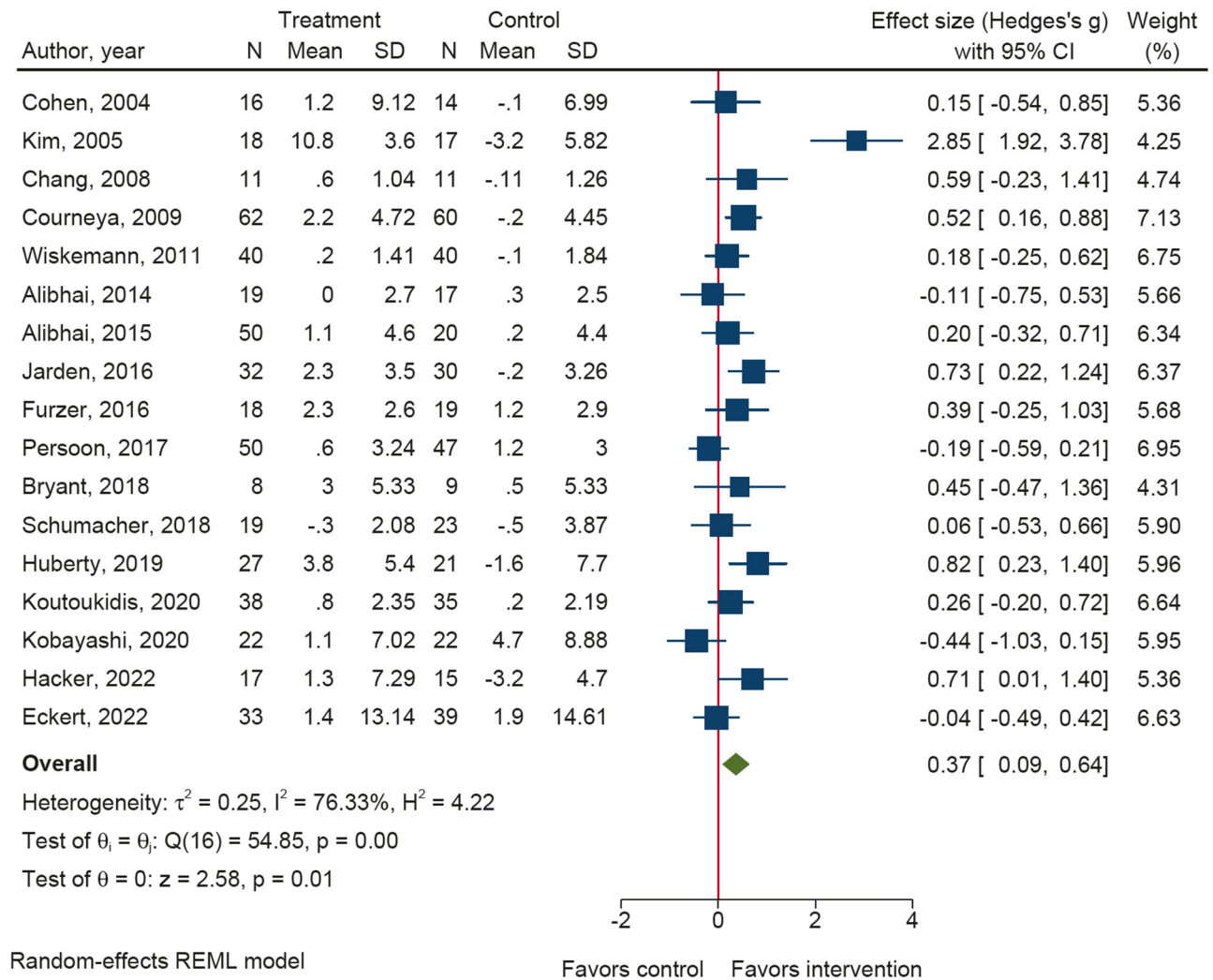

Supplement: Supplementary file 1 [file cancers-16-02962-s001.zip › Figure S17. Forest plot depicting the effects of exercise on depression.pdf]

Figure S18. Forest plot depicting the effects of exercise on fatigue.

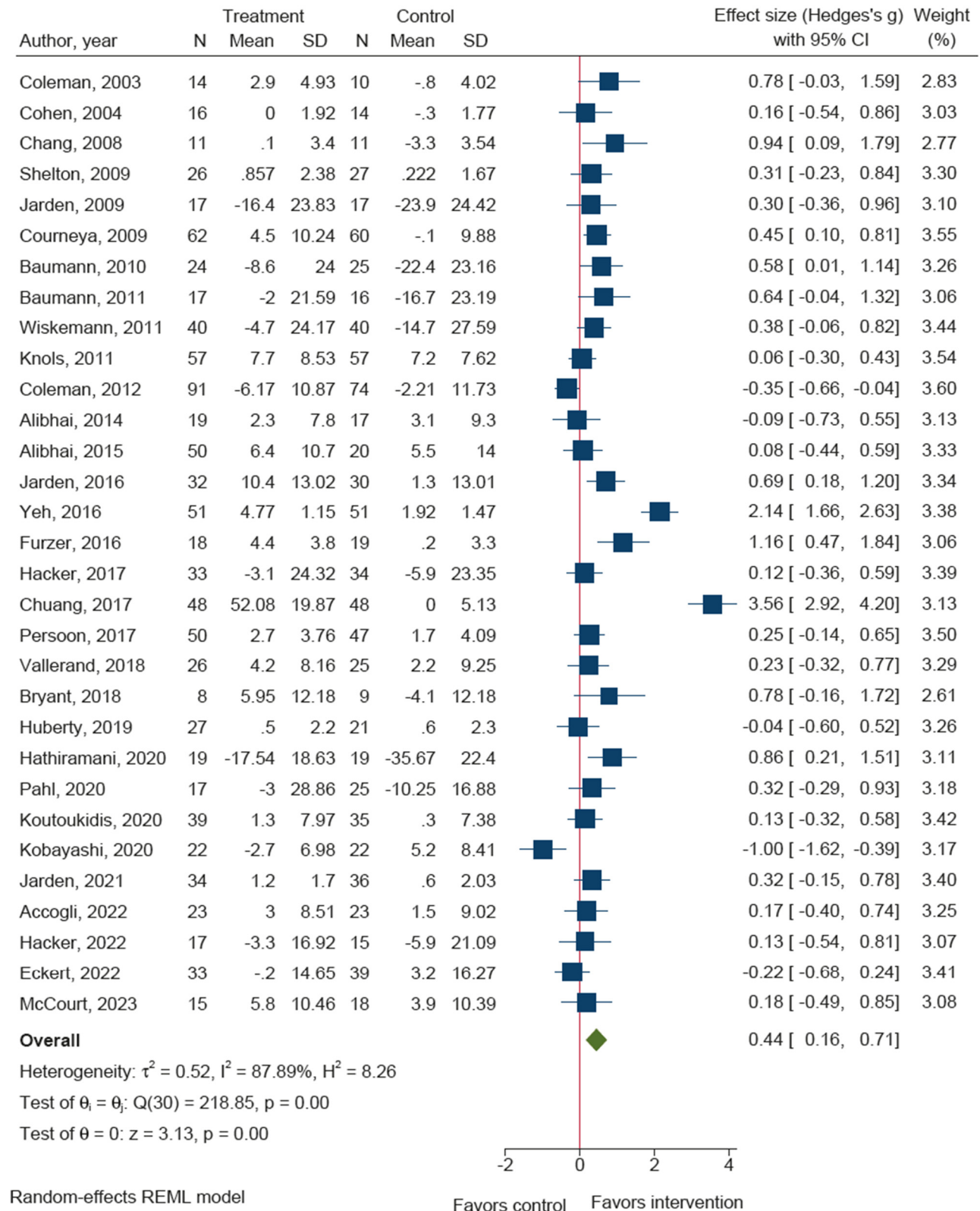

Supplement: Supplementary file 1 [file cancers-16-02962-s001.zip › Figure S18. Forest plot depicting the effects of exercise on fatigue.pdf]

Figure S19. Forest plot depicting the effects of exercise on pain.

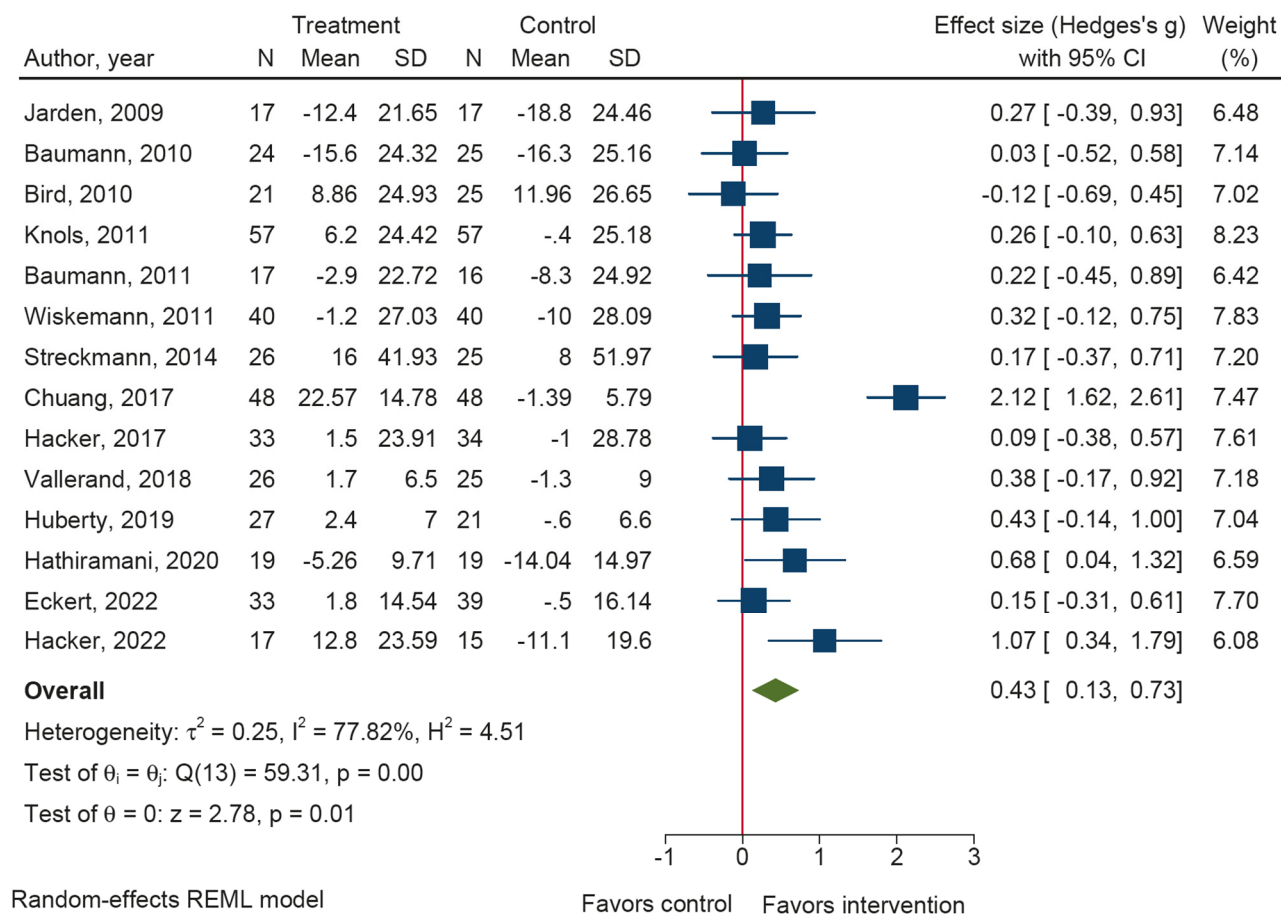

Supplement: Supplementary file 1 [file cancers-16-02962-s001.zip › Figure S19. Forest plot depicting the effects of exercise on pain.pdf]

Figure S21. Subgroup analyses on secondary outcome; aerobic capacity.

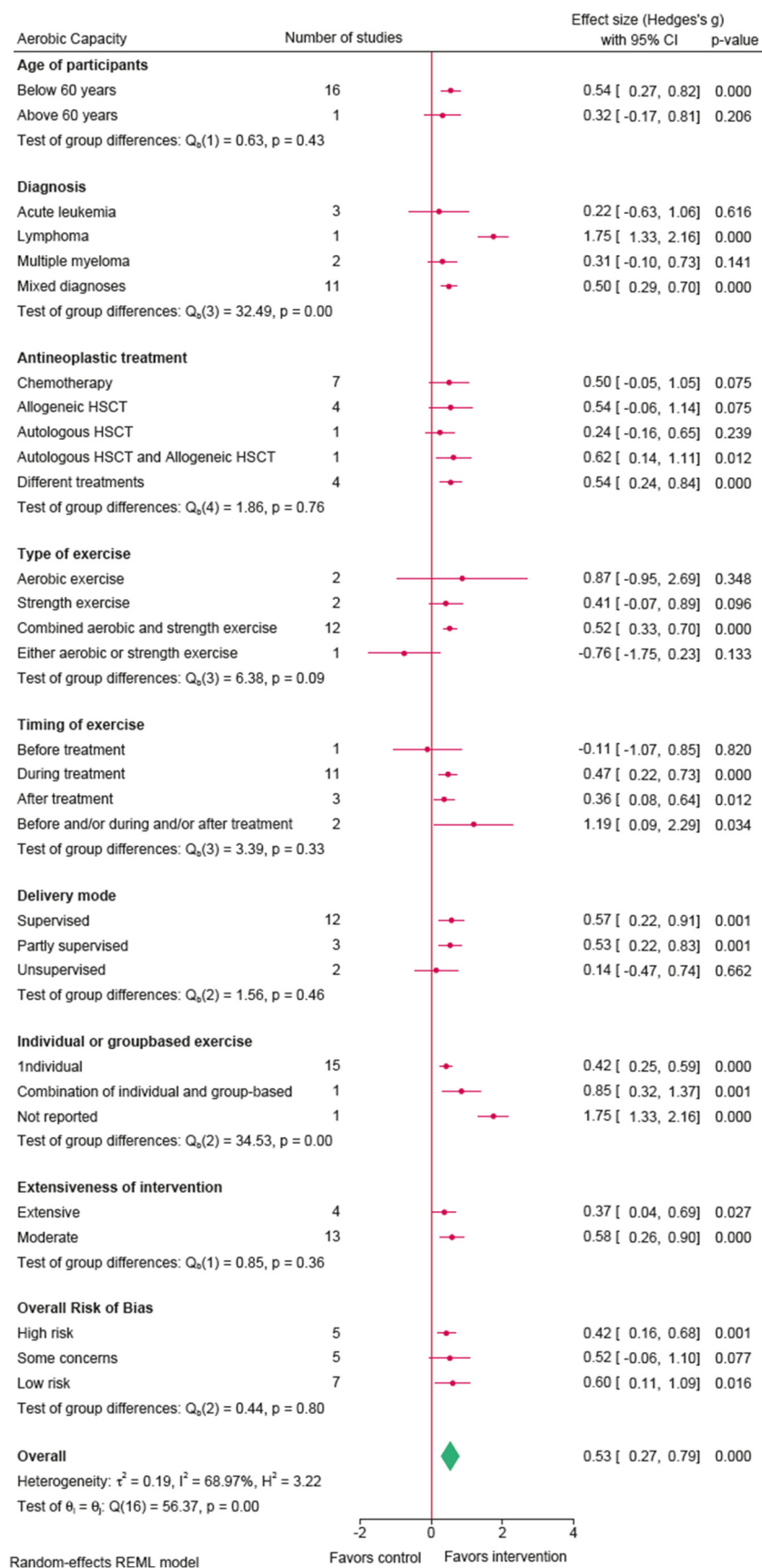

Supplement: Supplementary file 1 [file cancers-16-02962-s001.zip › Figure S21. Subgroup analyses on secondary outcome; aerobic capacity.pdf]

Figure S22. Subgroup analyses on secondary outcome; muscle strength.

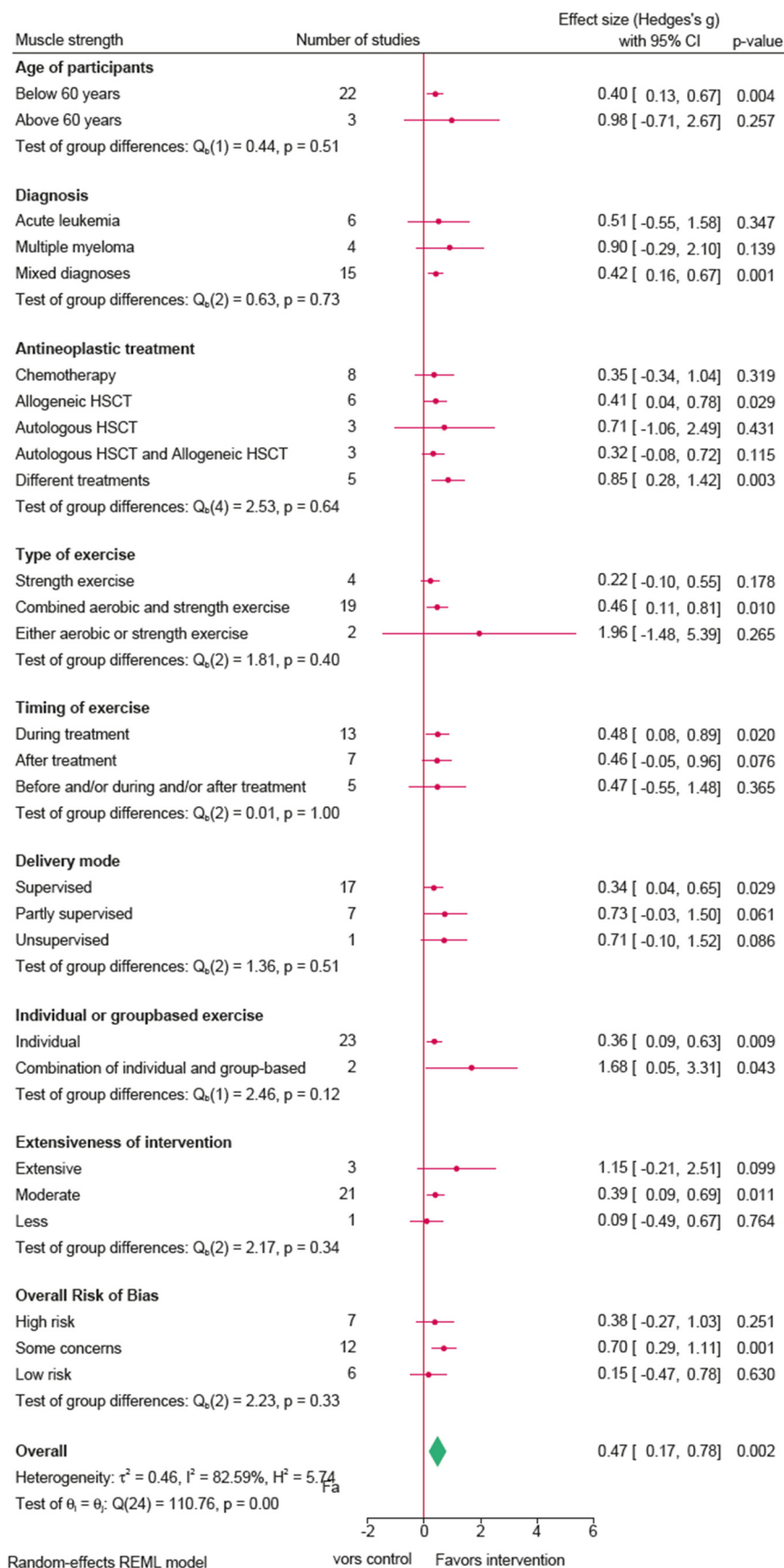

Supplement: Supplementary file 1 [file cancers-16-02962-s001.zip › Figure S22. Subgroup analyses on secondary outcome; muscle strength.pdf]

Figure S23. Subgroup analyses on secondary outcome; body composition.

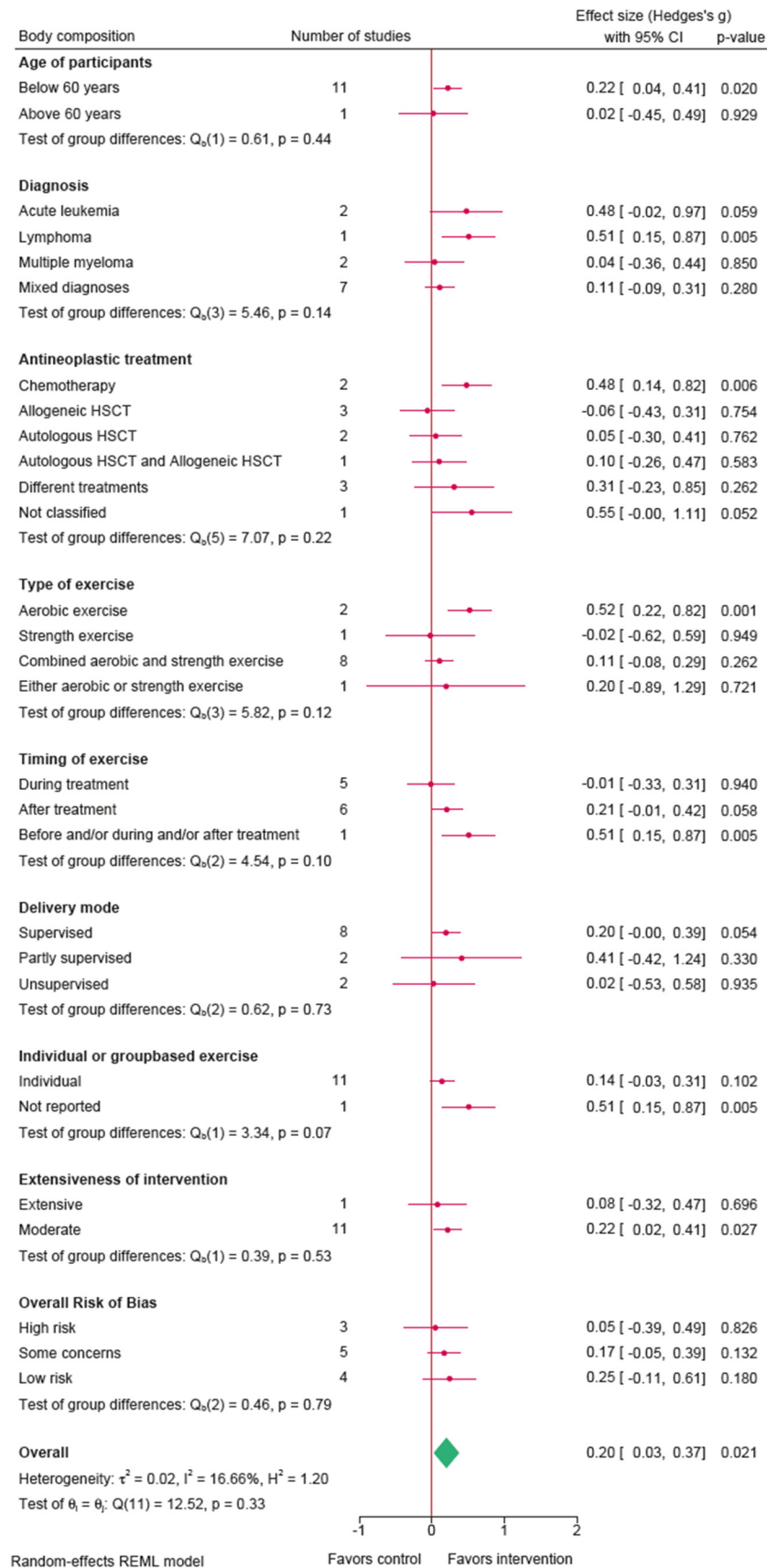

Supplement: Supplementary file 1 [file cancers-16-02962-s001.zip › Figure S23. Subgroup analyses on secondary outcome; body composition.pdf]

Figure S24. Subgroup analyses on secondary outcome; physical activity.

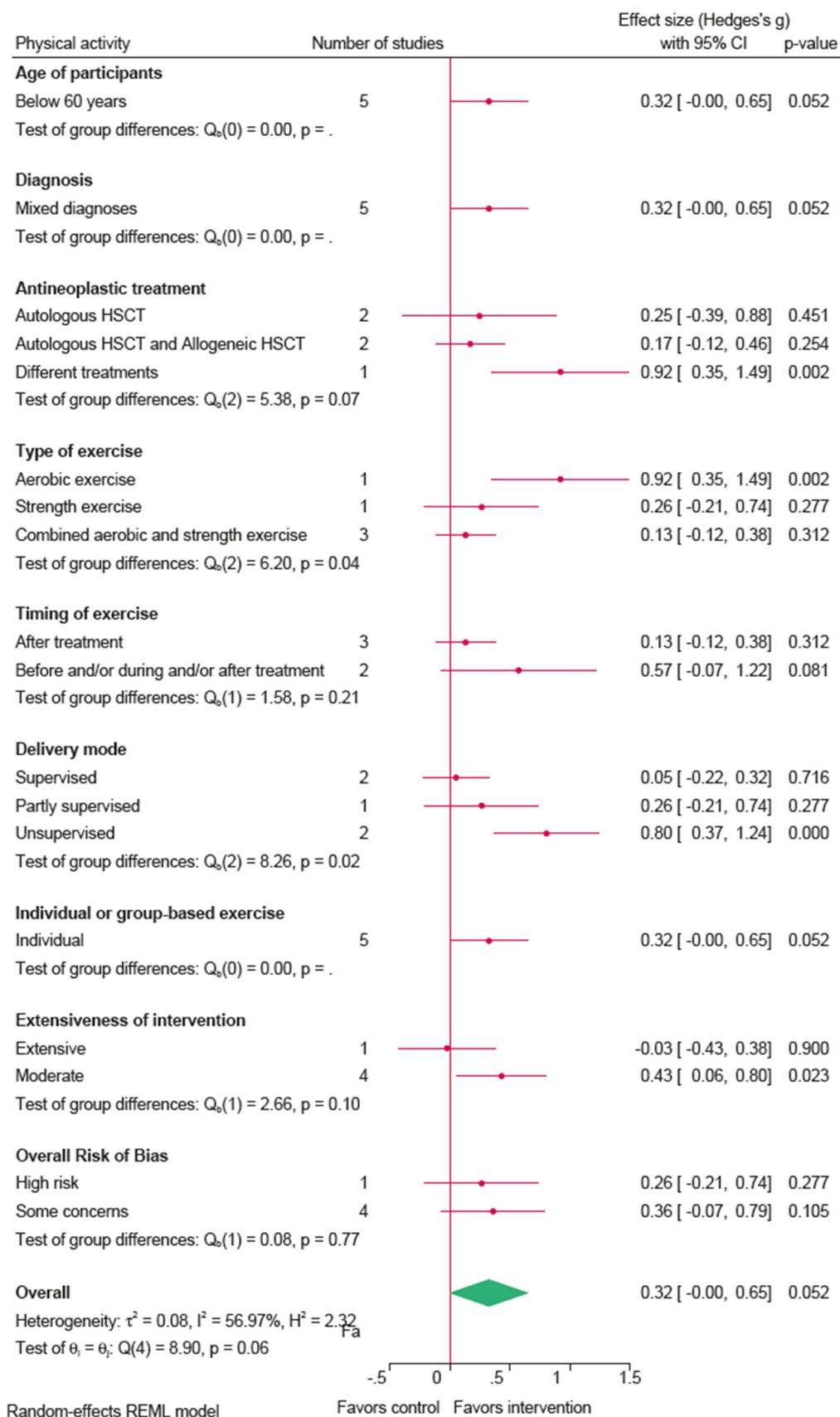

Supplement: Supplementary file 1 [file cancers-16-02962-s001.zip › Figure S24. Subgroup analyses on secondary outcome; physical activity.pdf]

Figure S25. Subgroup analyses on secondary outcome; QoL emotional.

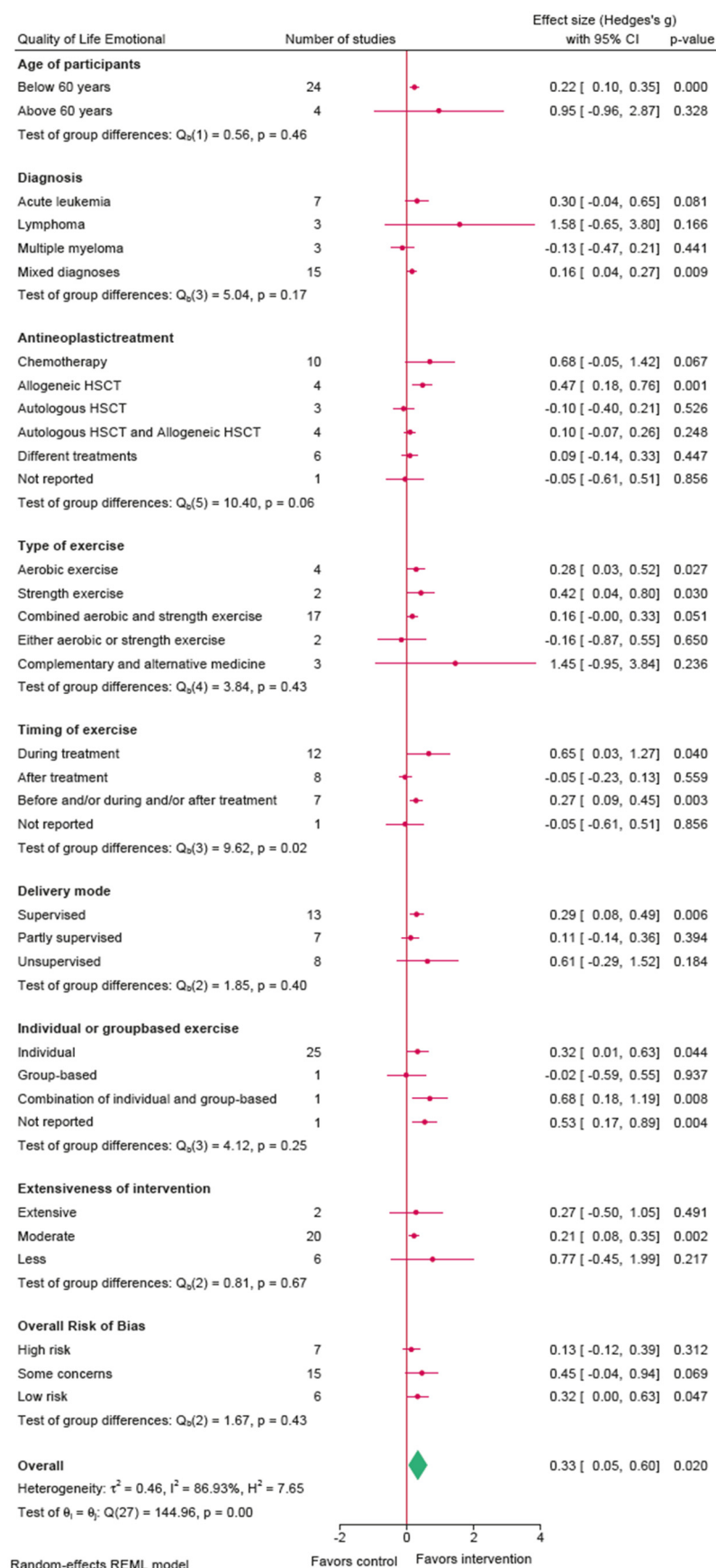

Supplement: Supplementary file 1 [file cancers-16-02962-s001.zip › Figure S25. Subgroup analyses on secondary outcome; QoL emotional.pdf]

Figure S26. Subgroup analyses on secondary outcome; QoL functional.

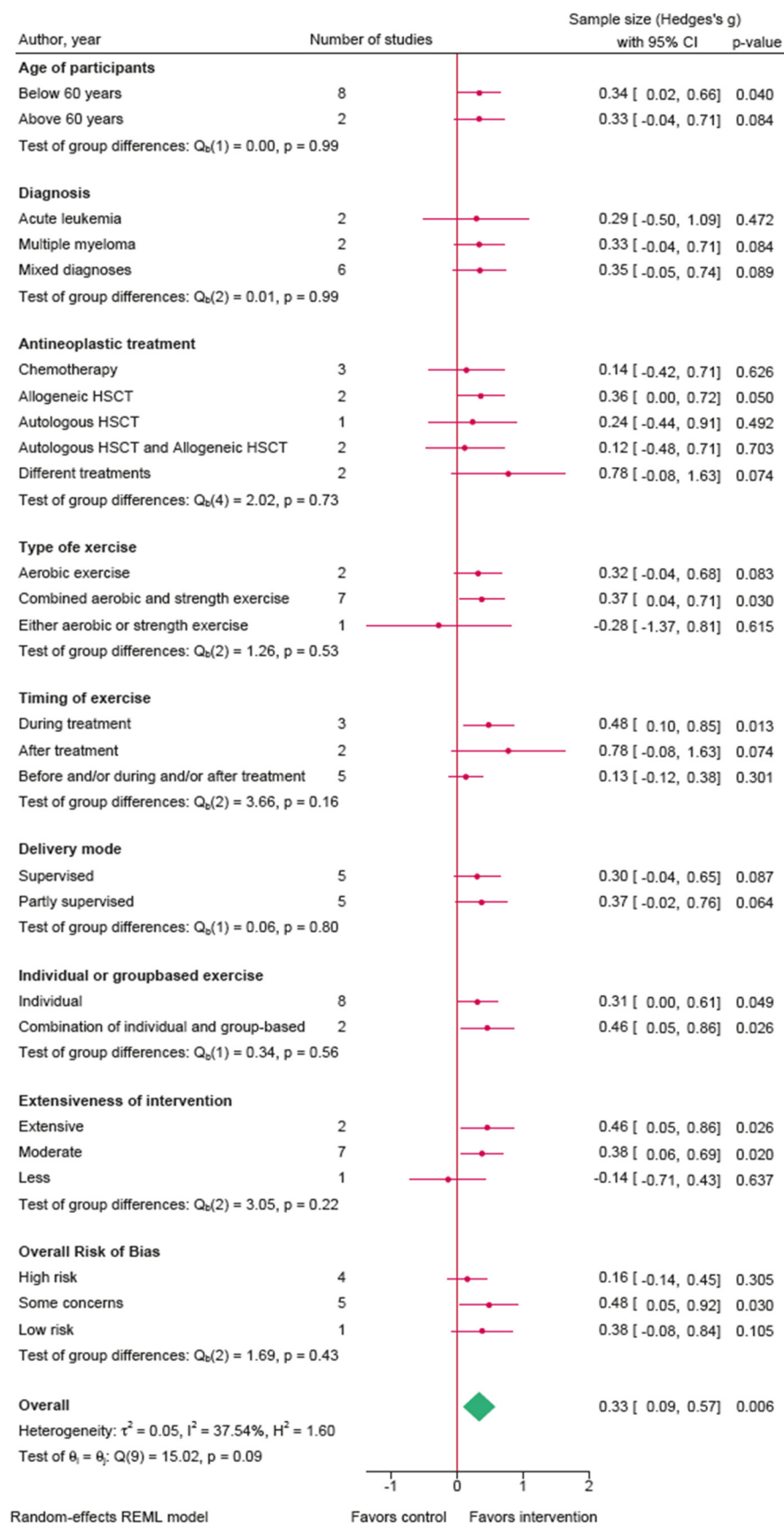

Supplement: Supplementary file 1 [file cancers-16-02962-s001.zip › Figure S26. Subgroup analyses on secondary outcome; QoL functional.pdf]

Figure S27. Subgroup analyses on secondary outcome; QoL physical.

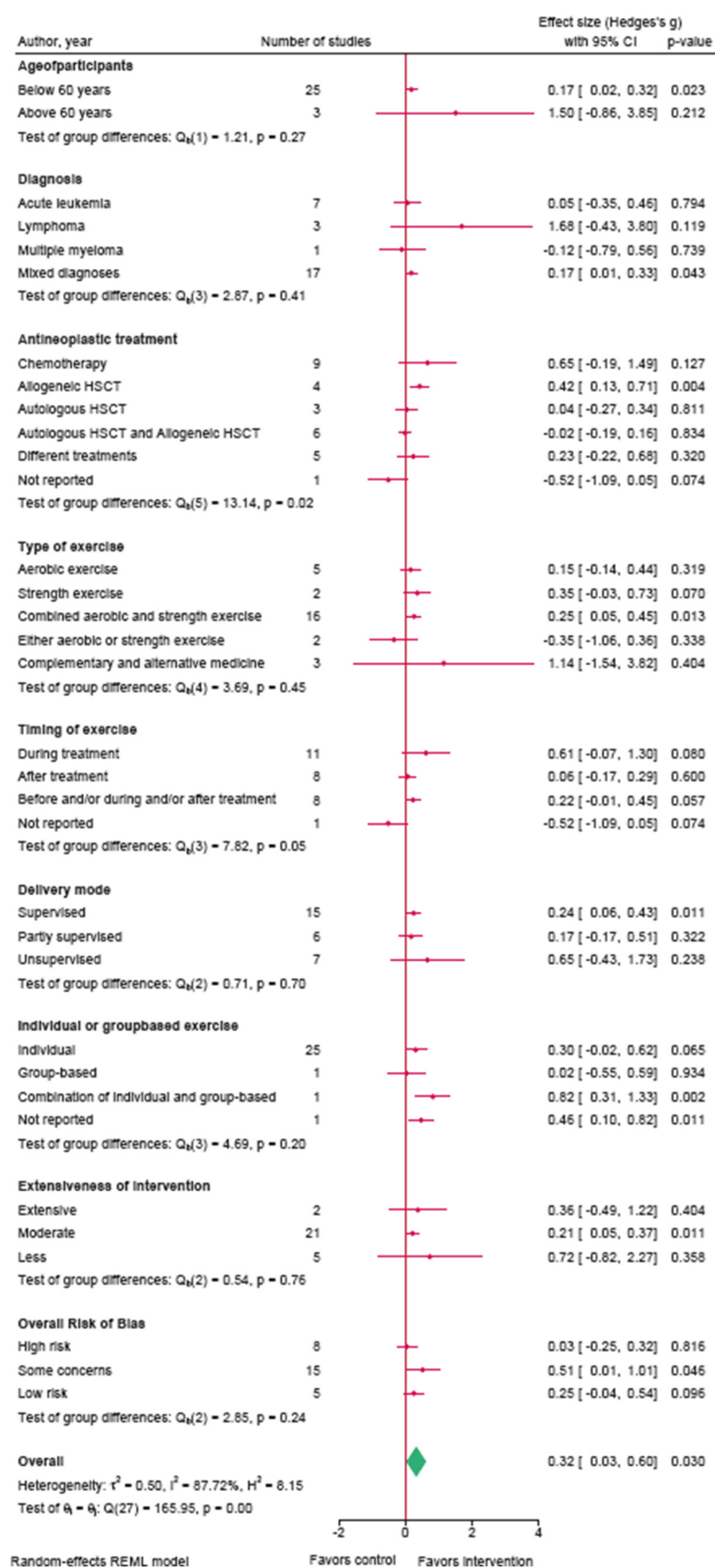

Supplement: Supplementary file 1 [file cancers-16-02962-s001.zip › Figure S27. Subgroup analyses on secondary outcome; QoL physical.pdf]

Figure S28. Subgroup analyses on secondary outcome; anxiety.

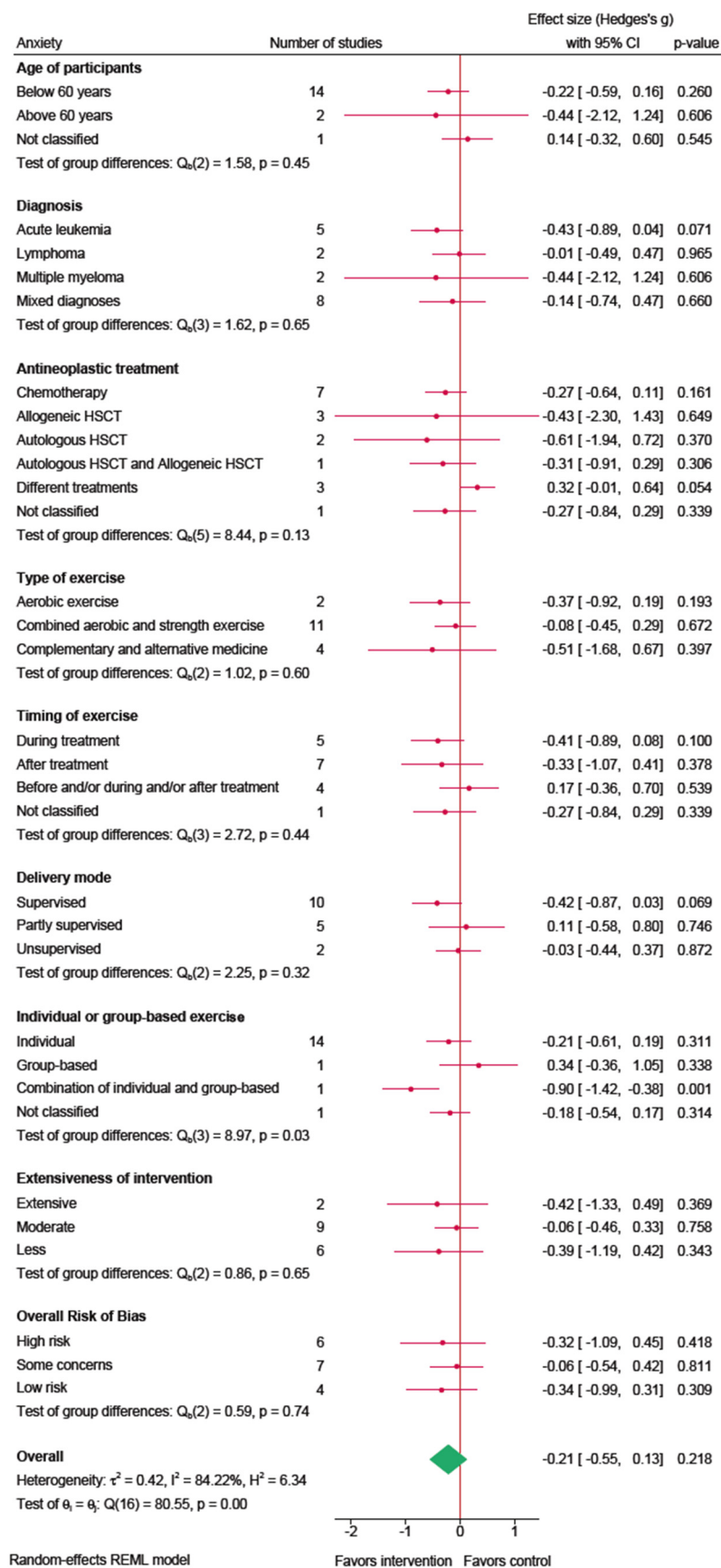

Supplement: Supplementary file 1 [file cancers-16-02962-s001.zip › Figure S28. Subgroup analyses on secondary outcome; anxiety.pdf]

Figure S29. Subgroup analyses on secondary outcome; depression.

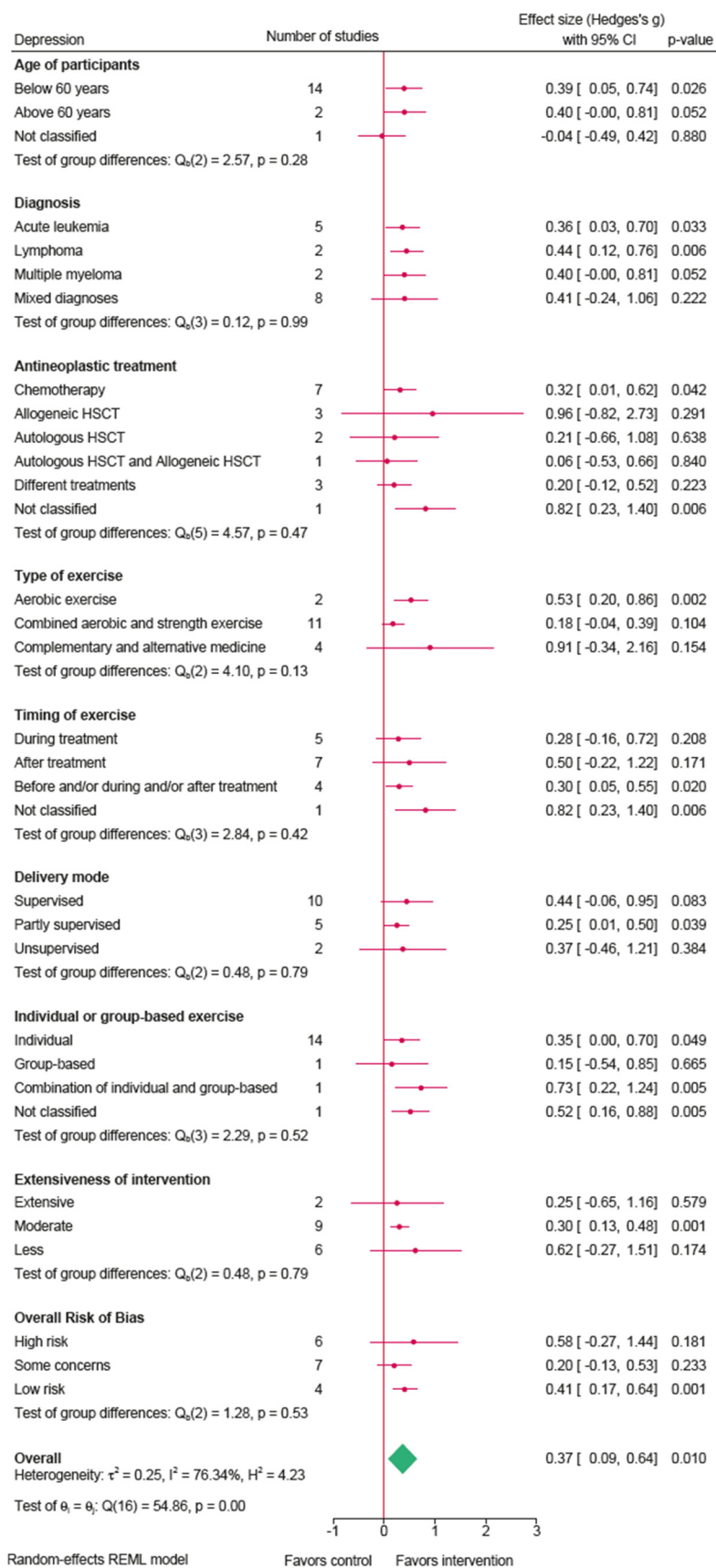

Supplement: Supplementary file 1 [file cancers-16-02962-s001.zip › Figure S29. Subgroup analyses on secondary outcome; depression.pdf]

Figure S30. Subgroup analyses on secondary outcome; fatigue.

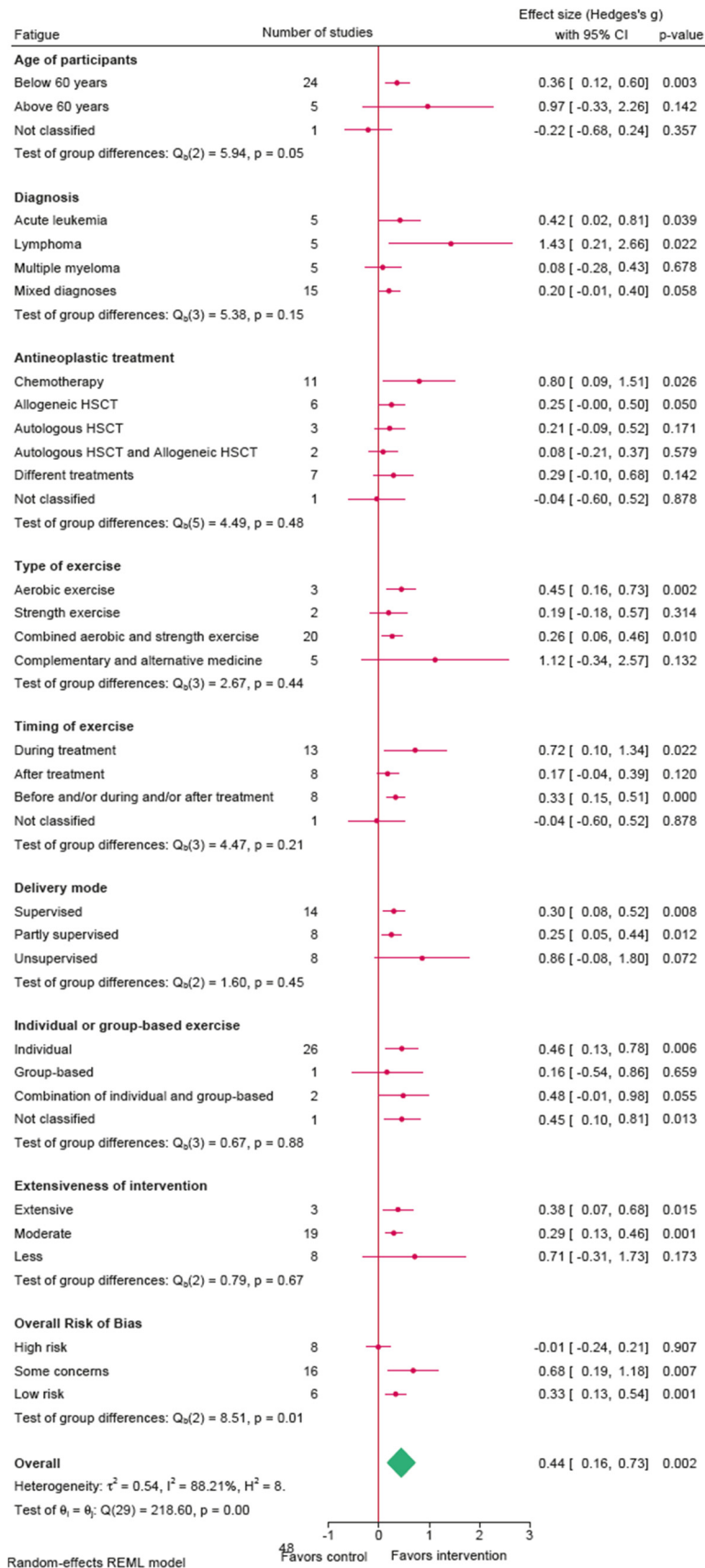

Supplement: Supplementary file 1 [file cancers-16-02962-s001.zip › Figure S30. Subgroup analyses on secondary outcome; fatigue.pdf]

Figure S31. Subgroup analyses on secondary outcome; pain.

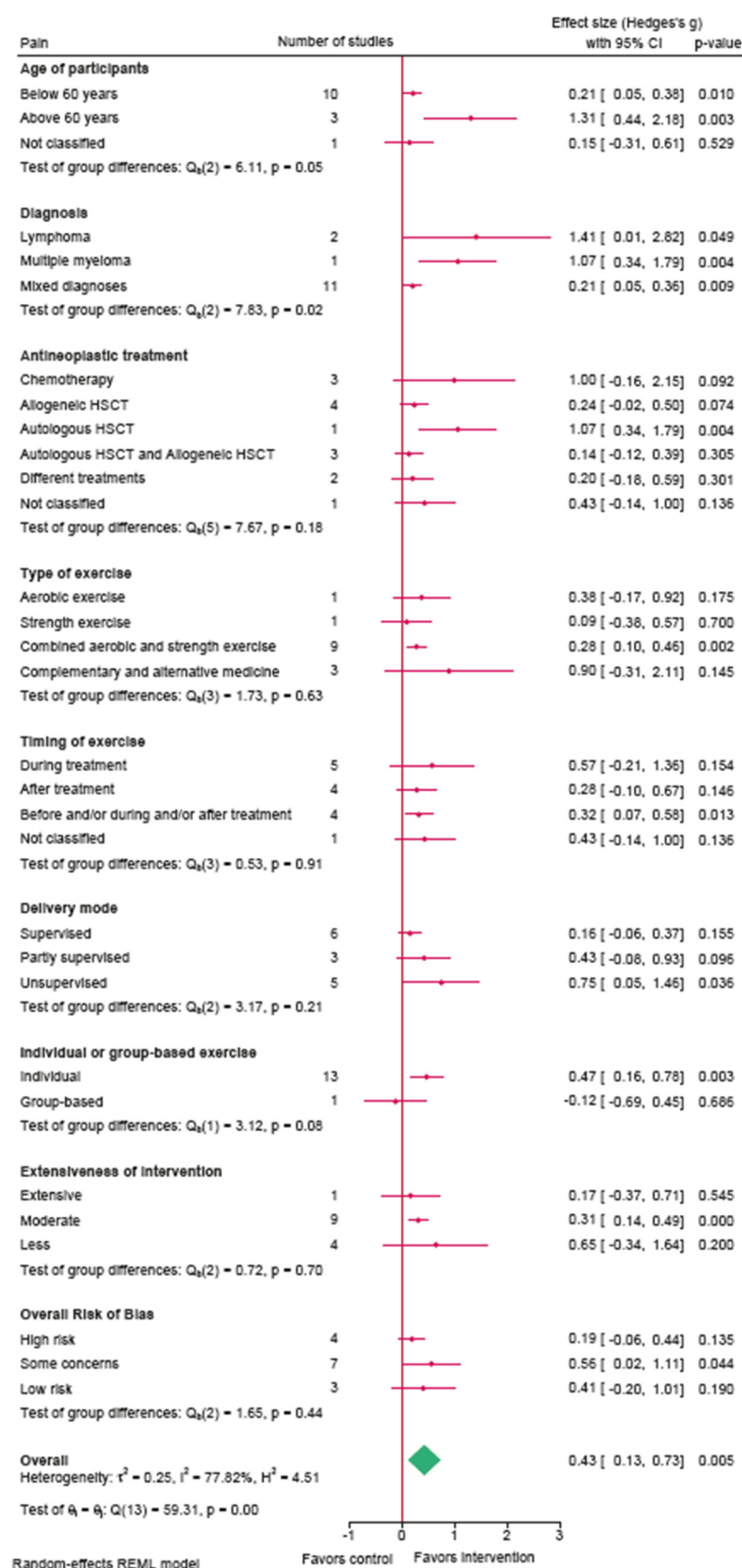

Supplement: Supplementary file 1 [file cancers-16-02962-s001.zip › Figure S31. Subgroup analyses on secondary outcome; pain.pdf]

Figure S4. Forest plot depicting the effects of exercise effects on physical function.

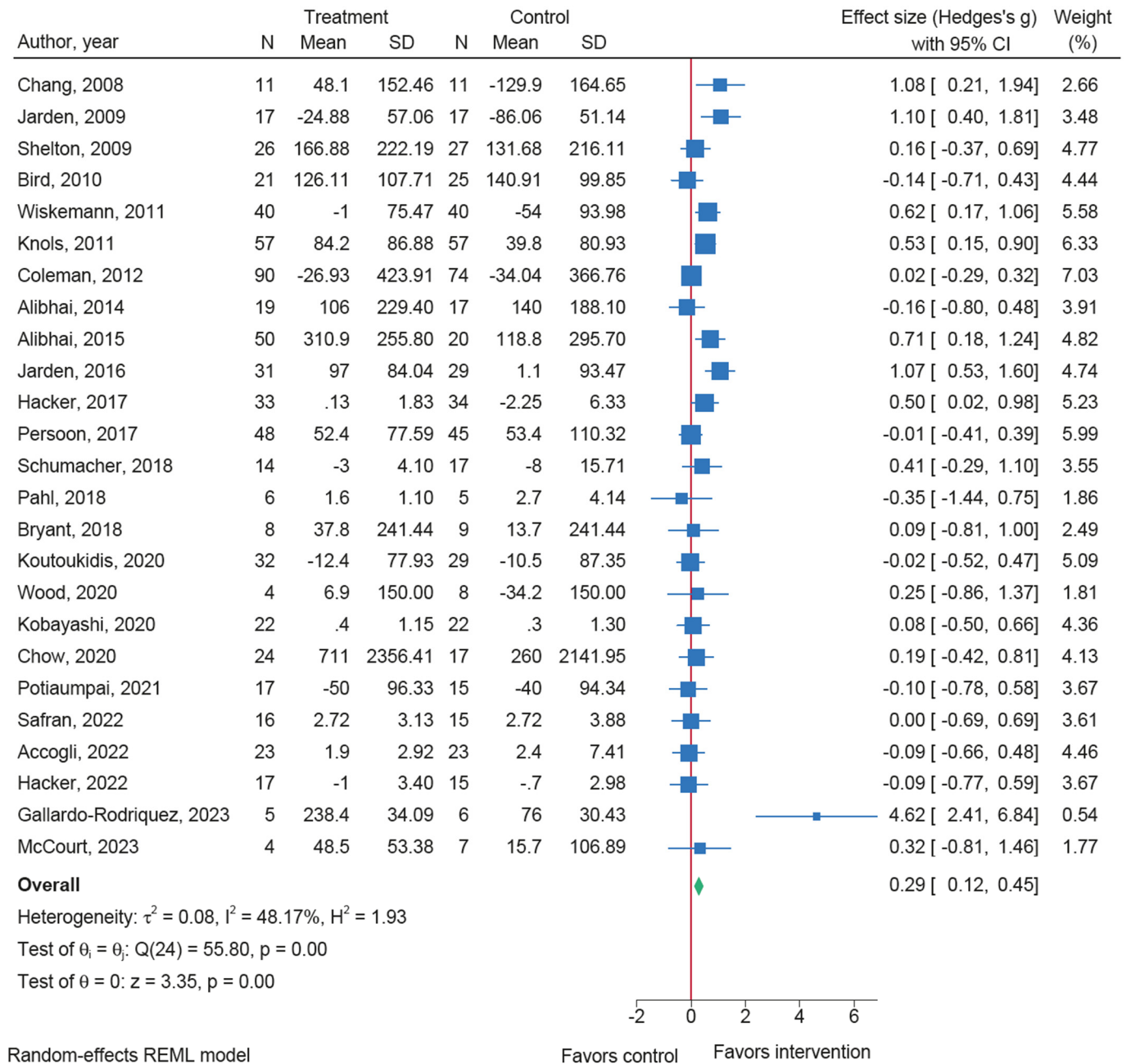

Supplement: Supplementary file 1 [file cancers-16-02962-s001.zip › Figure S4 Forest plot depicting the effects of exercise on physical function.pdf]

Figure S5. Forest plot depicting the effects of exercise effects on QoL global.

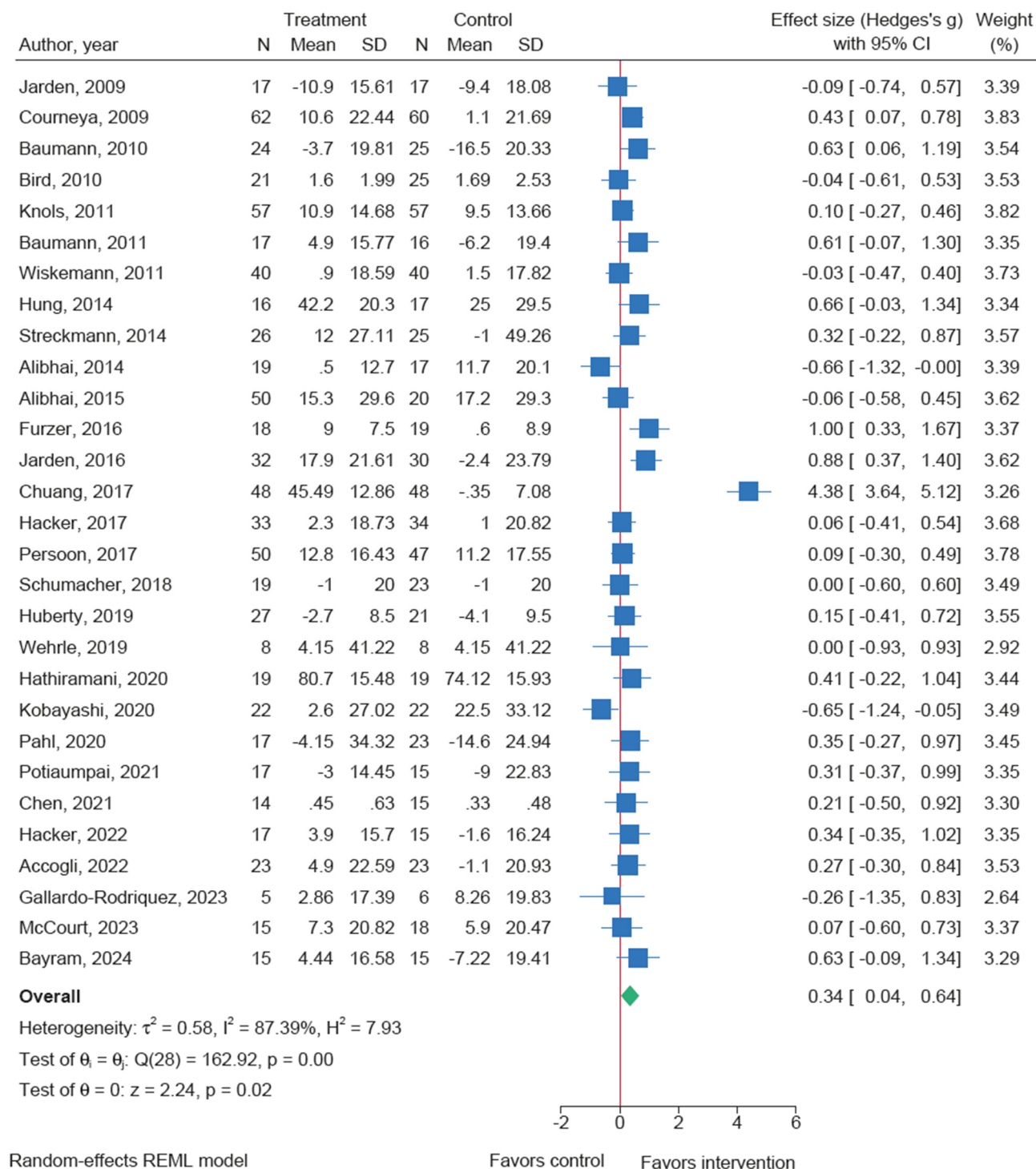

Supplement: Supplementary file 1 [file cancers-16-02962-s001.zip › Figure S5. Forest plot depicting the effects of exercise effects on QoL global.pdf]

Figure S8. Subgroup analyses on primary outcome; QoL global.

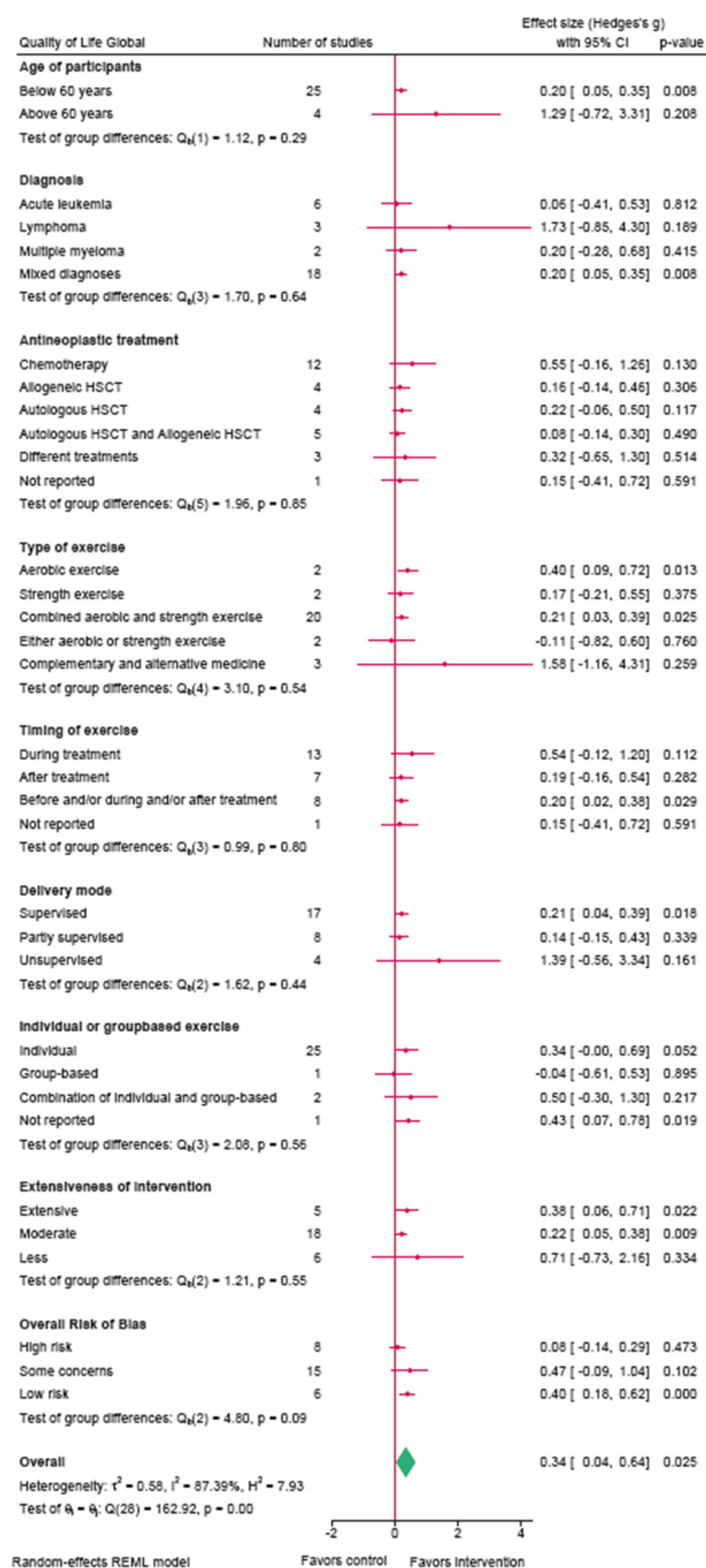

Supplement: Supplementary file 1 [file cancers-16-02962-s001.zip › Figure S8. Subgroup analyses on primary outcome; QoL global.pdf]

Figure S9. Forest plot depicting the effects of exercise effects on aerobic capacity.

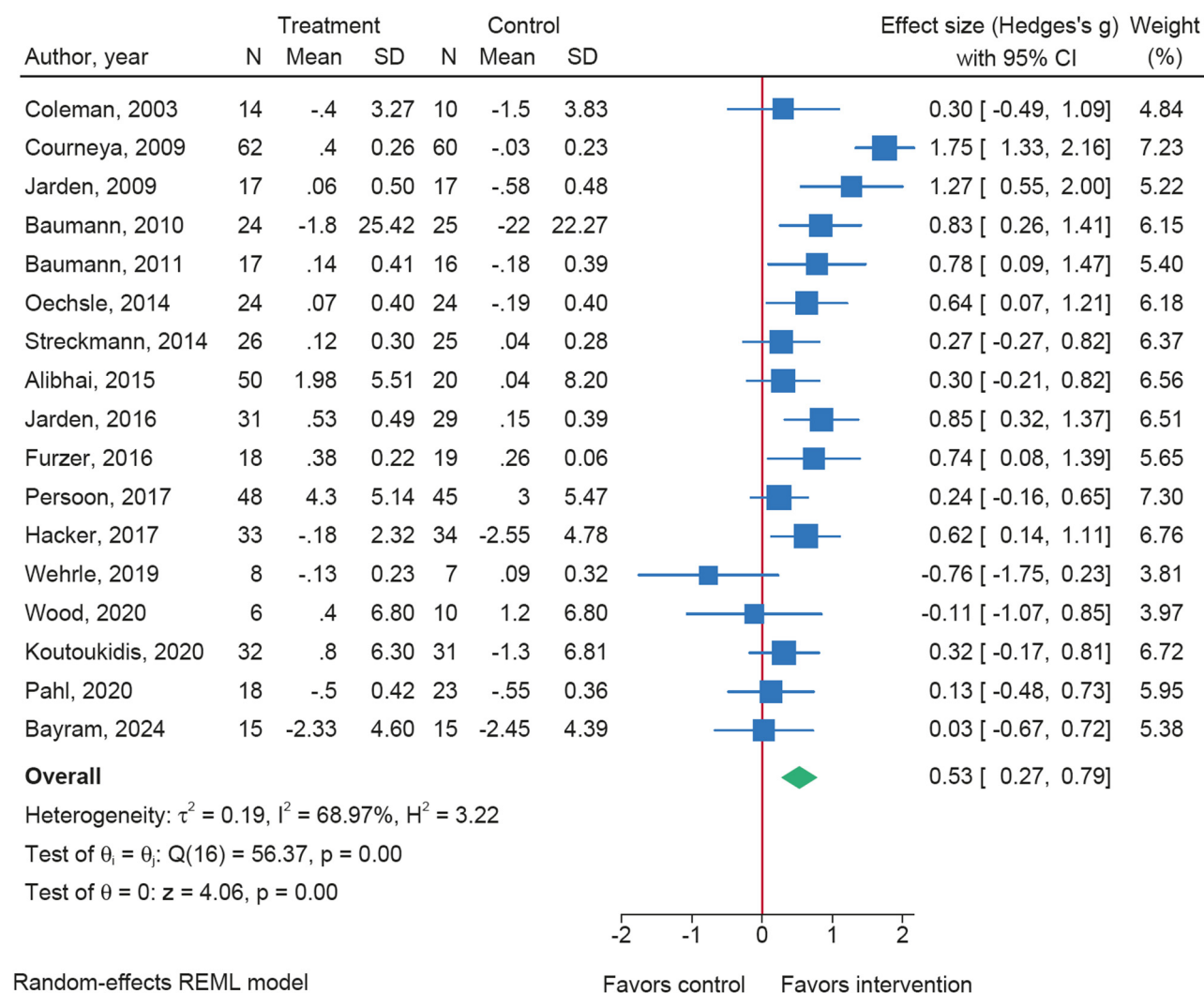

Supplement: Supplementary file 1 [file cancers-16-02962-s001.zip › Figure S9. Forest plot depicting the effects of exercise effects on aerobic capacity.pdf]
